# In Silico and In Vitro Studies of the Approved Antibiotic Ceftaroline Fosamil and Its Metabolites as Inhibitors of SARS-CoV-2 Replication

**DOI:** 10.3390/v17040491

**Published:** 2025-03-28

**Authors:** Cássia Delgado, Pablo Andrei Nogara, Milene Dias Miranda, Alice Santos Rosa, Vivian Neuza Santos Ferreira, Luisa Tozatto Batista, Thamara Kelcya Fonseca Oliveira, Folorunsho Bright Omage, Flávia Motta, Izabela Marques Bastos, Laura Orian, João Batista Teixeira Rocha

**Affiliations:** 1 Departamento de Bioquímica e Biologia Molecular, Centro de Ciências Naturais e Exatas, Universidade Federal de Santa Maria, Santa Maria 97000-000, RS, Brazil; cassia.delgado@acad.ufsm.br (C.D.); joao.rocha@ufsm.br (J.B.T.R.); 2 Instituto Federal de Educação, Ciência e Tecnologia Sul-rio-grandense (IFSul), Bagé 96400-000, RS, Brazil; 3 Laboratório de Morfologia e Morfogênese Viral, Instituto Oswaldo Cruz, Fundação Oswaldo Cruz, Rio de Janeiro 21041-250, RJ, Brazil; alicerosa@aluno.fiocruz.br (A.S.R.); vivian.ferreira@ioc.fiocruz.br (V.N.S.F.); luisatozbatista@gmail.com (L.T.B.); thamarafonseca@ufmg.br (T.K.F.O.); 4 Programa de Pós-Graduação em Biologia Celular e Molecular, Instituto Oswaldo Cruz, Fundação Oswaldo Cruz, Rio de Janeiro 21041-250, RJ, Brazil; 5 Biological Chemistry Laboratory, Department of Organic Chemistry, Institute of Chemistry, University of Campinas (UNICAMP), Campinas 13000-000, SP, Brazil; omage.folorunsho@acad.ufsm.br; 6 Laboratório de interface patógeno-hospedeiro, Departamento de Biologia Celular, Universidade de Brasília (UnB), Brasília 70910-900, DF, Brazil; fnmotta@unb.br (F.M.); dourado@unb.br (I.M.B.); 7 Dipartimento di Scienze Chimiche, Università degli Studi di Padova, Via Marzolo 1, 35129 Padova, Italy; laura.orian@unipd.it; 8 Departamento de Bioquímica, Instituto de Ciências Básicas da Saúde, Universidade Federal do Rio Grande do Sul (UFRGS), Porto Alegre 90000-000, RS, Brazil

**Keywords:** cysteine proteases, drug repurposing, ceftaroline fosamil, viral replication inhibition, SARS-CoV-2, molecular docking, molecular dynamics

## Abstract

The SARS-CoV-2 proteases M^pro^ and PL^pro^ are critical targets for antiviral drug development for the treatment of COVID-19. The 1,2,4-thiadiazole functional group is an inhibitor of cysteine proteases, such as papain and cathepsins. This chemical moiety is also present in ceftaroline fosamil (CF), an FDA-approved fifth-generation cephalosporin antibiotic. This study investigates the interactions between CF, its primary metabolites (M1 is dephosphorylated CF and M2 is an opened β-lactam ring) and derivatives (protonated M1H and M2H), and its open 1,2,4-thiadiazole rings derivatives (open-M1H and open-M2H) with SARS-CoV-2 proteases and evaluates CF’s effects on in vitro viral replication. *In silico* analyses (molecular docking and molecular dynamics (MD) simulations) demonstrated that CF and its metabolites are potential inhibitors of PL^pro^ and M^pro^. Docking analysis indicated that the majority of the ligands were more stable with M^pro^ than PL^pro^; however, *in vitro* biochemical analysis indicated PL^pro^ as the preferred target for CF. CF inhibited viral replication in the human Calu-3 cell model at submicromolar concentrations when added to cell culture medium at 12 h. Our results suggest that CF should be evaluated as a potential repurposing agent for COVID-19, considering not only viral proteases but also other viral targets and relevant cellular pathways. Additionally, the reactivity of sulfur in the 1,2,4-thiadiazole moiety warrants further exploration for the development of viral protease inhibitors.

## 1. Introduction

The coronaviruses (CoVs) are enveloped viruses with a positive-sense, single-stranded RNA genome that encodes large replicase polyproteins that are processed by viral peptidases to generate proteins involved in viral replication. Understanding the life cycle of the virus is critical to reveal their pathogenic potential [1,2,3,4,5,6,7].

Cysteine proteases are one of the four main groups of peptide-bond hydrolases. These enzymes use a thiolate anion (R–S⁻) of a cysteine (Cys) side chain as the nucleophile in the hydrolysis of peptide bond [2,3]. The cysteinyl proteases are found in all forms of life and can also be encoded by single-stranded RNA viruses. In vertebrates, cysteinyl proteases can mediate a wide variety of physiological and pathological processes. In different viruses, these proteases play key roles in virion formation, release, and entry into the host cells. Typically, cysteinyl proteases metabolize the formation of critical viral proteins inside host cells [4,5,8].

The SARS-CoV-2 papain-like protease (PL^pro^) and the 3C chymotrypsin-cysteine-like peptidase or main protease (M^pro^) post-translationally process the viral pp1a and pp1ab polyproteins into non-structural proteins (nsp). The cysteinyl residues found in the catalytic moieties of the M^pro^ and PL^pro^ are directly involved in the hydrolysis of specific peptide bonds presented in the large polyproteins pp1a and pp1ab [2,9,10].

The M^pro^ of coronaviruses is encoded by the nsp5 gene. Upon infection, M^pro^ cleaves pp1a at 11 conserved sites to generate 12 active nsps (nsp4 to nsp16), which are essential for the replication and synthesis of viral subgenomic RNAs [2,11]. PL^pro^ is encoded by the nsp3 gene and cleaves the viral pp1a at three conserved sites between nsp1 and nsp4, generating the active nsp1, nsp2, and nsp3. Like M^pro^, PL^pro^ is crucial for the formation of the viral replication/transcription complex (RTC). In addition to activating viral nsps, PL^pro^ can also cleave host ubiquitinated proteins and the ISG15 protein, thereby directly interfering with host metabolism in virus-infected cells [2,12].

In PL^pro^, the catalytic site is composed of a triad (Cys111, His272, and Asp286), while M^pro^ features a catalytic dyad (Cys145 and His41). These cysteine residues are directly involved in the hydrolysis of specific peptide bonds within the large polyproteins pp1a and pp1ab [11,13,14]. Consequently, the cysteine proteases of coronaviruses (MERS-CoV, SARS-CoV, and SARS-CoV-2) have become key targets for repurposing existing therapeutics and developing novel agents [14,15,16,17,18,19,20,21,22,23,24].

Since the beginning of the pandemic, substantial efforts have been dedicated to finding inhibitors for SARS-CoV-2 proteases [15,17,18,25,26,27,28]. For example, organochalcogen compounds such as ebselen, disulfiram, and tideglusib have demonstrated the ability to inhibit SARS-CoV-2 M^pro^ *in vitro*. Additionally, ebselen and diphenyl diselenide have shown efficacy in inhibiting viral replication in human cells [27,29,30]. The inhibitory properties of some of these molecules have also been analyzed and validated *in silico* [14,19,28,31,32].

**Figure 1:**
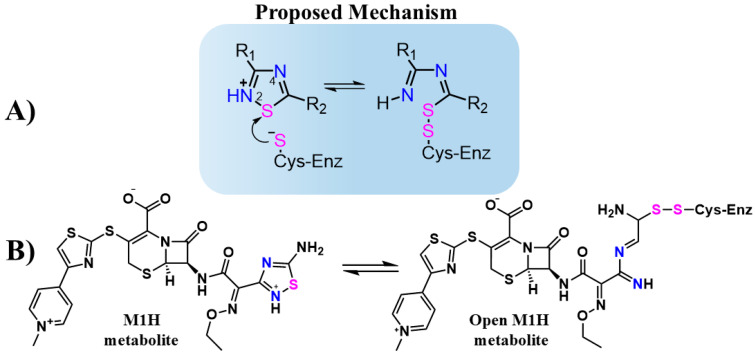
(**A**) Structure and proposed inhibitory mechanism of cysteine enzymes (Cys-Enz) by 1,2,4-thiadiazoles molecules. R1 and R2 are organic groups. (**B**) Proposed inhibitory mechanism of Cys-Enz by CF metabolite (M1H) through covalent bond formation (S-S). The 1,2,4-thiadiazole heteroatoms Sulfur (pink) and Nitrogen (blue) are highlighted to emphasizes the proposed mechanism before and after the heterocycle opening.

Jin et al. evaluated thousands of molecules and identified tideglusib as the only organochalcogen compound-containing 1,2,4-thiadiazole group that inhibits the M^pro^ with an IC_50_ of 1.55 μM [27]. Although the inhibitory mechanism of tideglusib against M^pro^ has not been thoroughly investigated in detail, the 1,2,4-thiadiazole moiety is known to inhibit cysteinyl proteases such as papain and cathepsins B, L, and K [33,34,35]. This inhibition is attributed to the sulfur atom in the 1,2,4-thiadiazole ring, which acts as an electrophilic center. The thiolate (S-) group of the cysteinyl proteases attacks the sulfur atom of the thiadiazole ring, forming a disulfide bond and triggering ring opening (Figure 1A) [34,35,36].

**Figure 2:**
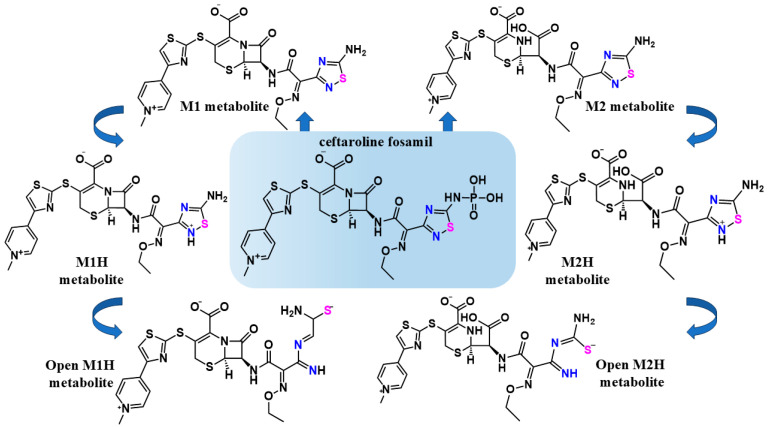
The chemical structures of the antibiotic CF and its metabolic scheme evaluated in this work. The structures of CF, its M1 metabolite, and its M2 metabolite were obtained from the DrugBank database [43]. The structures of M1H and M2H metabolites, were used to support the proposed mechanism for the inhibition of cysteine proteases by the 1,2,4-thiadiazole functional group [35]. The structures of open-M1H and open-M2H metabolites were determined, taking into account the proposed mechanism for the inhibition of cysteine proteases by the 1,2,4-thiadiazole functional group, after disruption of the aromatic ring and the interaction with the active sites. The 1,2,4-thiadiazole heteroatoms Sulfur (pink) and Nitrogen (blue)_are highlighted to emphasizes the proposed mechanism before and after the heterocycle opening.

Previously, Kumar et al. [37] performed an *in silico* study on potential repurposing drugs in the Korea Chemical Bank drug reuse database (KCB-DR). They have indicated ceftaroline fosamil (CF) (Figure 2) as a putative inhibitor of SARS-CoV M^pro^. CF is an approved antibiotic for clinical use against a wide range of Gram-positive and Gram-negative bacteria. CF is part of a new generation of cephalosporins, broad-spectrum β-lactams effective against methicillin-resistant Staphylococcus aureus (MRSA) [38,39,40,41]. Developed as an intravenous prodrug, it is converted into the M1-metabolite (Figure 2) through enzymatic dephosphorylation in the human body [38,39,40,41]. In the liver, ceftaroline is metabolized by esterase enzymes, resulting in the opening of the β-lactam ring, forming the M2 metabolite (Figure 2) [39,40]. CF and its dephosphorylated ceftaroline metabolites (M1 and M2) (Figure 2) have the prototypal 1,2,4-thiadiazole functional group.

The simulations by Kumar et al. [37] demonstrated the interaction between CF and the active site of M^pro^ by hydrogen bonding with key residues, such as His41 [37]. Based on these results, Delgado et al. conducted *in silico* studies to assess the interaction of FDA-approved therapeutic agents containing the 1,2,4-thiadiazole functional group, including CF, with SARS-CoV-2 proteases [42]. Using virtual screening from the DrugBank database [43], rigid molecular docking, molecular dynamics (MD) simulations, and density functional theory (DFT) calculations, they confirmed the predicted interaction of CF and its metabolites with the active sites of M^pro^ and PL^pro^. Based on previous studies and DFT results, they proposed a potential inhibition mechanism in which the 1,2,4-thiadiazole group inhibits cysteine proteases through heterocycle opening, enabling covalent binding between the thiolate of the cysteine protease and the sulfur atom of the 1,2,4-thiadiazole group (Figure 1A) [34,42]. Building on these findings and the favorable DFT energy values of the proposed binding mechanism, this study aimed to analyze distance simulations and intermolecular interactions involving CF, its metabolites, and the structures resulting from thiadiazole ring cleavage as potential targets of SARS-CoV-2 proteases (Figure 1B).

Pharmacokinetic studies indicated that intravenous administration of therapeutic doses of CF resulted in micromolar range plasma and epithelial lining fluid (ELF) concentrations of the M1 metabolite [44]. CF was used in patients hospitalized with COVID-19 in Italy; however, the analysis focused on its use in controlling bacterial co-infections and did not provide data on severity or mortality in COVID-19 patients [45].

In order to validate the *in silico* data and the potential of repurposing CF and the dephosphorylated M1 and M2 metabolites against SARS-CoV-2, we carried out *in vitro* studies. The potential inhibitory effects of CF on recombinant M^pro^ and PL^pro^ enzymes and against the SARS-CoV replication in Calu-3 cells were investigated. Detailed *in silico* studies with CF, its primary metabolites (M1 and M2), its protonated derivatives (M1H and M2H), and its open rings derivatives (open-M1H and open-M2H) (Figure 2) were performed to determine their interaction with the proteases M^pro^ and PL^pro^.

Although theoretical rationale and *in silico* studies pointed to a potential interaction of ceftaroline (CF) with the viral proteases M^pro^ and PL^pro^, our *in vitro* tests using infected Calu-3 cells with two different MOIs (0.01 and 0.1), treated with 12 h pulses over a 48 h post-infection period, revealed significant antiviral activity. We observed that the EC_50_ values of CF against viral replication were lower than the IC_50_ observed for the isolated proteases, suggesting that CF’s antiviral mechanism may involve interactions with other viral proteins or cellular components beyond the specific proteases. This type of multimodal activity is not uncommon among antiviral compounds. For example, remdesivir acts by inhibiting viral RNA polymerases but also exhibits effects on modulating the immune response [46]. Another example is favipiravir, which, in addition to inhibiting viral replication through interaction with RNA polymerases, appears to interfere with host metabolic processes that affect viral replication [47,48,49]. Therefore, our findings reinforce the need to explore additional potential mechanisms through which CF may exert its antiviral activity.

## 2. Materials and Methods

### 2.1. Ceftaroline Fosamil Antibiotic, Metabolites, and Derivatives

The DrugBank database www.godrugbank.com (accessed on 18 September 2023) was utilized to obtain the FDA-approved antibiotic CF and its primary metabolites: the dephosphorylated metabolite of CF is termed the M1 metabolite and the dephosphorylated metabolite that had its β-lactam ring opened is termed the M2 metabolite (Figure 2) [43]. The structures of the M1H and M2H metabolites were used in view of the proposed mechanism for the inhibition of cysteine proteases by the 1,2,4-thiadiazole functional group [34,50]. The structures of the open-M1H and open-M2H metabolites were prepared referring to the proposed mechanism for the inhibition of cysteine proteases by the 1,2,4-thiadiazole functional group. Specifically, the nucleophilic attack of the cysteinyl residues from M^pro^ or PL^pro^ at the electrophilic S in the 1,2,4-thiadiazole functional group is followed by the formation of a disulfide S-S bond between the catalytic cysteinyl residues in the two enzymes with the S atom of thiadiazole group, (resulting in the opening of thiadiazole ring. The aromatic ring was then disrupted, and subsequently, the disulfide S-S bond was reduced by a second cysteinyl residue, releasing the open metabolite. Avogadro 1.0.2 version software was used to create and refine them.

### 2.2. Docking Simulations

The Auto Dock Vina program was used for the docking simulations [51], with an exhaustiveness of 50, according to a previous study [19]. Different docking simulation methodologies were applied to explore the common and most probable binding poses of the ligands, simulating the macromolecular target in its natural/cellular environment). One rigid and one semiflexible docking simulation was performed with the M^pro^ active site, CF, and the M1, M2, M1H, M2H, open-M1H, and open-M2H metabolites. One rigid and one semiflexible docking simulation was performed with PL^pro^, using the same configurations of the ligands described above. The M^pro^ and PL^pro^ crystallographic structures were obtained from the Protein Data Bank (PDB) with the codes 7CWB and 7JN2, respectively. For each molecule, the 20 best conformers (in terms of ∆G) were analyzed in the Discovery Studio Visualizer program [14,52]. Two conformers were chosen, i.e., the conformer with the largest negative binding energy [53] and the conformer with the shortest S···S interaction (between protein and ligand) (Table 1). The conformers of CF, its metabolites, and its derivatives which displayed the best interaction with the Cys residues of the active sites in M^pro^ and PL^pro^ (in terms of S···S distances and ∆G) were highlighted (Table 2). Specifically, the distance between the S atom (ligand) and the S atom (Cys) was considered an indicator of potential covalent bond formation between 1,2,4-thiadiazole compounds and metabolites and the enzymes.

#### 2.2.1. Rigid Simulations Focusing on the Active Site with Cys and His Charged

M^pro^ and PL^pro^ docking simulations were performed considering the active site in the ionic/charged state, i.e., Cys was deprotonated and His was protonated (and Asp286 was deprotonated for PL^pro^), to evaluate the binding poses and intermolecular interactions obtained in the catalytic site and surrounding amino acids of the two enzymes. The grid boxes were placed in the same configuration previously described. The M^pro^ grid box of size 25 × 35 × 25 Å was centered on the active site of chain A (−14.04, 17.44, 66.22), and for PL^pro^, the grid box was centered on the active site (39.64, 30.68, 1.66; size: 20 × 20 × 20 Å), according to previous studies [14,19]. Water, ions, ligands, and other molecules were removed from the protein structures; then, the hydrogen atoms were added using the CHIMERA program, and 100 steps of energy minimization were carried out [54].

#### 2.2.2. Semi-Flexible Docking Simulations with Cys and His Charged

Auto Dock Vina 1.2.0 was used for flexible docking simulations in M^pro^ and PL^pro^ active sites, according to the previous protocol [55]. Two flexible docking simulations were analyzed, one for the M^pro^ monomer (M^pro^ (Cys145 and His41 with flexible lateral chains) and one for PL^pro^ (Cys111, His272, and Asp286 with flexible lateral chains). In both cases, the Cys amino acids were in the thiolate (S^−^) form, and the histidine was protonated. The hydrogens atoms of these specific amino acids were removed, and then using the CHIMERA program, 100 steps of energy minimization were carried out [54].

### 2.3. Molecular Dynamics Simulations

The selected structures obtained in the docking analyses were utilized for molecular dynamics simulations. In both enzymes, the largest negative ∆G conformer of the rigid docking simulations focusing on the active site Cys and His charged was utilized (Table 1. ^a^M^pro^–^b^PL^pro^). Hydrogen atoms were added to the poses using the “h_add” command in PyMOL prior to force-field generation with Antechamber and the frcmod module of the Amber suite. For the PL^pro^ system, the conformer of the M1H metabolite was selected with a charge of +1, while the conformer of the M2H metabolite had a charge of 0. In the case of the M^pro^ system, the open-M1H and open-M2H metabolites were selected with a charge of −1.

The simulation of M^pro^ and PL^pro^ was performed using the AMBER20 version software suite [56]. For the protein components, the ff14SB force field was employed [57]. Ligands, including both closed- and open-ring metabolites, were parameterized using the GAFF2 force field, facilitating the generation of precise bond, angle, and dihedral parameters critical for simulation within the Amber framework. In the case of PL^pro^, the unique zinc finger motif was modeled using the ZAFF parameter set, providing a tailored approach to capture the specific interactions involving the zinc ion. The treatment of activated cysteine residues as CYM and protonated histidine in the active site as HIP was in line with established protocols for simulating M^pro^ and PL^pro^ [32], ensuring consistency and reliability in the representation of these critical active site features. The solvation of the protein–ligand complexes was executed by enclosing them in a truncated octahedral box filled with TIP3P water molecules. A buffer distance of 12 Å from the solute to the box edge was maintained, ensuring sufficient hydration of the system and minimizing boundary effects. To achieve electrostatic neutrality and replicate the physiological ionic conditions, an appropriate number of Na^+^ ions were added to the M^pro^ system, while the PL^pro^ net charge of zero was maintained. This step is essential to establish an ionic strength that mirrors the intracellular milieu, thus enhancing the biological relevance of the simulation results.

The GPU (CUDA) version of the PMEMD module of AMBER20 version was used for all the simulations on NVIDIA GeForce RTX 3070 Ti. The equilibration procedure was executed for 50 ns prior to the 100 ns production run in order to guarantee that the systems were properly equilibrated. Prior to the equilibration procedure, a minimization protocol consisting of three steps was followed. During the initial 5000 steps of minimization, water molecules were restricted with a force constant of 10 kcal/mol/Å^2^. During the subsequent phase, the system as a whole was limited to 5000 steps of solute reduction under the same force constant constraint. In the third and last stage, the whole system was minimized for an additional 5000 steps without the constraint using conjugate and steepest descent methods.

PCA was performed on the backbone atoms of the aligned trajectories to identify the principal modes of motion. It was conducted using the PCA class in MD Analysis [58] which decomposes the covariance matrix of atomic displacements to yield principal components. The first two principal components, capturing the most significant motions, were plotted for each simulation to visualize the conformational landscape explored by the proteins.

### 2.4. SARS-CoV-2 PL^pro^ and M^pro^ Expression and Purification

The recombinant SARS-CoV-2 M^pro^ and PL^pro^ were produced as previously described with some modifications [59]. Briefly, the SARS-CoV-2 PL^pro^ gene was cloned into the pET-19b expression vector and SARS-CoV-2 M^pro^ was cloned into pGEX-6P-1. Both plasmids were transformed into the *Escherichia coli* strain BL21(DE3). The expression of the SARS-CoV-2 PL^pro^ recombinant protein was induced by the addition of 0.1 mM IPTG and 1.0 mM ZnCl_2_ for 18 h at 18 °C. SARS-CoV-2 M^pro^ recombinant protein expression was induced by 1 mM IPTG for 18 h at 18 °C. To purify the recombinant enzymes, cells were harvested, lysed with BugBuster^®^ (Merck Millipore, Burlington, MA, USA), and centrifuged at 16,000× *g* for 20 min at 4 °C. The supernatant was submitted to affinity chromatography on a nickel–agarose resin (Sigma, Burlington, MA, USA). After the resin had been extensively washed with 50 mM Tris-HCl (pH 8.0), 0.5M NaCl, and 5 mM imidazole, bound recombinant enzyme was eluted with the same buffer containing 80 mM imidazole. The purified enzyme was dialyzed against 20 mM Tris-HCl (pH 7.3), concentrated, and stored at −80 °C (PL^pro^) or 4 °C (M^pro^).

#### Ceftaroline Fosamil SARS-CoV-2 PL^pro^ and M^pro^ Inhibition Assay

The IC_50_ values were determined from 3.125 to 400 μM CF plus the control. Inhibition assays were performed, in triplicate, with 52 nM of the recombinant PL^pro^ protein from SARS-CoV-2 or 100 nM of the recombinant M^pro^ from SARS-CoV-2 in 20 mM Tris-HCl (pH 7.3) and 1 mM EDTA for 15 min. The substrate LKGG-AMC was added to a 100 μL reaction mixture at a final concentration of 20 μM for PL^pro^. For M^pro^, the substrate Dabcyl-Lys-Thr-Ser-Ala-Val-Leu-Gln-Ser-Gly-PheArg-Lys-Met-Glu(Edans)-NH2 was used also at 20 μM in a 100 μL reaction mixture.

Subsequently, AMC-released fluorescence was measured under excitation at 355 nm and emission at 460 nm in kinetic mode for 20 min using the SpectraMax^®^ M5 Microplate Reader (Thermo Fisher Scientific, Waltham, MA, USA). The curves were fitted in a four-parameter logistic (4 PL) nonlinear regression model using GraphPad Prism^®^ 8 (version 8.0.2) software for the calculation of IC_50_ [59].

### 2.5. In Vitro Study in Calu-3 and Vero E6 Cell Models

#### 2.5.1. Cell Culture and Virus

African green monkey kidney cells (Vero, subtype E6, ATCC CRL-1586) and human type II pneumocyte model cells (Calu-3 generously provided by the Farmanguinhos platform RPT11M) were cultured and maintained in high-glucose Dulbecco’s Modified Eagle Medium (DMEM; Gibco™, Waltham, MA, USA), supplemented with 1% penicillin/streptomycin (Gibco™, Waltham, MA, USA), HEPES buffer (Gibco™, Waltham, MA, USA), and 10% fetal bovine serum (FBS; Gibco™, Waltham, MA, USA). The cultures were incubated at 37 °C in a humidified atmosphere containing 5% CO_2_. The cells were seeded in 96-well plates (Kasvi, São José dos Pinhais, PR, Brazil) at a density of 1.0 × 10^4^ cells/well for Vero E6 and 1.5 × 10^4^ cells/well for Calu-3 and maintained in an incubator at 37 °C with 5% CO_2_. After seeding, the cell plates were processed for various experimental assays. The SARS-CoV-2 B.1 lineage virus (GenBank #MT710714, SisGen AC58AE2) was stored at −80 °C. All procedures involving SARS-CoV-2 cultures were conducted in a biosafety level 3 (BSL-3) laboratory, following the guidelines of the World Health Organization (WHO) [60,61].

Calu-3 cells (1.5 × 10^4^ cells/well) were cultured in a 96-well plate (Kasvi, São José dos Pinhais, PR, Brazil) and treated with a concentration gradient of CF (0.8, 1.6, 3.1, 6.3, 12.5, 25, 50, 100, and 200 µM) for 72 h at 37 °C in a 5% CO_2_ atmosphere. CF was initially dissolved in 100% dimethyl sulfoxide (DMSO; Nova Biotecnologia, Cotia, SP, Brazil) to achieve a final concentration of 0.1% (*v*/*v*) upon dilution in DMEM, ensuring no adverse effects on Calu-3 cell growth or metabolism [62]. Following the treatment period, cytotoxicity was assessed using methylene blue staining to determine the percentage of viable cells [63]. Post-treatment, the cells were washed with saline solution, fixed, and stained for 1 h at 37 °C in 5% CO_2_ with a methylene blue solution (Hank’s balanced salt solution (HBSS), 0.6% methylene blue, and 1.25% glutaraldehyde). After staining, the solution was removed, and the plate was washed and dried at room temperature. An elution solution (49% PBS, 50% ethanol, and 1% acetic acid) was added, and the plate was incubated for 15 min at room temperature. The supernatant of the stained cultures was then transferred to a new 96-well plate and analyzed at a wavelength of 660 nm using a spectrophotometer (Loccus LMR-96i-4, São Paulo, SP, Brazil). The 50% cytotoxic concentration (CC_50_) of CF was determined by comparing the treated cells to the untreated control cells [64].

#### 2.5.2. SARS-CoV-2 Replication Inhibition Assay

The inhibition of viral replication was assessed in monolayers of Calu-3 cells (1.5 × 10^4^ cells/well) infected with SARS-CoV-2 at a multiplicity of infection (MOI) of 0.01 or 0.1 for 1 h at 37 °C in a 5% CO_2_ atmosphere. Subsequently, the cells were treated with a semi-log concentration gradient of CF (0.1, 0.316, 1, 3.16, and 10 µM) for 12, 24, 36, and 48 h. At 12 h intervals, 20 µL of the supernatants was collected and stored at −80 °C for virus titer determination. To maintain the initial concentration, a new pulse of treatment with the same volume was added to the cells, completing the treatment at 48 h. For virus titration, Vero E6 cells at a density of 1.0 × 10^4^ cells/well were incubated with the harvested supernatants at various dilutions (1:100 to 1:12,800) for 1 h at 37 °C in a 5% CO_2_ atmosphere. Following this incubation, 50 µL of carboxymethylcellulose medium (CMC) at 2.4% (DMEM 10x, sodium bicarbonate 0.22%, FBS 2%, penicillin 1%, and streptomycin 1%) was added to each well, and the cells were cultured for 72 h under the same conditions. The cells were then fixed with 4% formalin for 3 h, washed, and stained with 0.04% crystal violet for 1 h [63]. Viral titers were determined by counting plaque-forming units (PFU) and expressed as PFU/mL.

#### 2.5.3. Statistical Analysis

All graphs were generated and analyzed using GraphPad Prism software version 10.2.1. The data presented represent the average values obtained from at least three replicates. The concentration of molecules necessary to achieve 50% effective inhibitory activity (EC_50_) and the cytotoxic concentration resulting in a 50% loss of cell viability (CC_50_) were determined by analyzing the best-fit curves (R^2^ ≥ 0.9) using nonlinear regression of the inhibitor’s logarithm versus the normalized response. The selectivity index (SI), defined as the ratio of CC_50_ to EC_50_, represents the safety margin for the *in vitro* use of the molecule.

## 3. Results and Discussion

### 3.1. Docking Analysis

**Figure 3:**
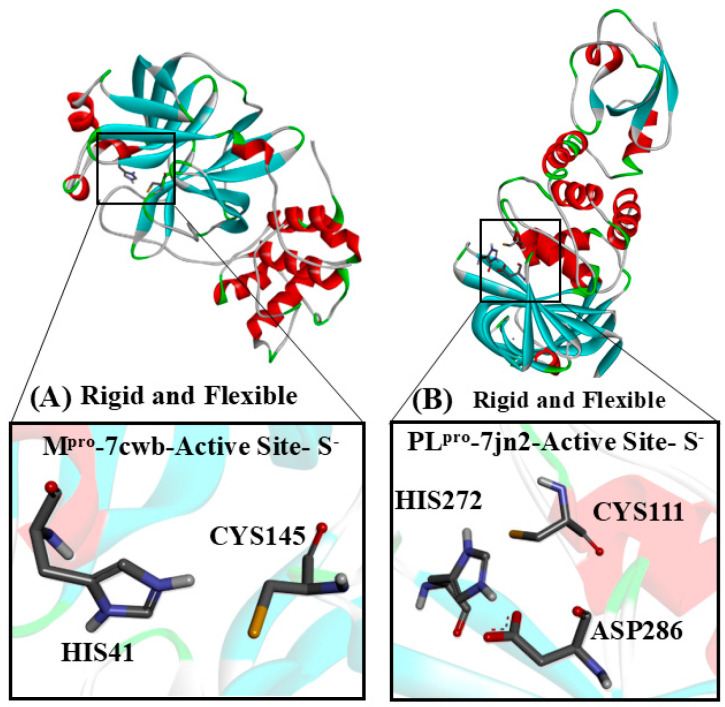
The target cysteinyl residues in the SARS-CoV-2 M^pro^ active site (Cys145) and PL^pro^ active site (Cys111) are shown. (**A**) The M^pro^ monomer, with Cys and His residues, deprotonated and protonated, respectively. (**B**) PL^pro^, with His and Cys residues of the active site, protonated and deprotonated, respectively.

Different docking simulation strategies were applied to explore the common and most probable binding poses of the analyzed compounds, aiming to use the macromolecular target in its natural/cellular environment (Figure 3). Here, we will present the results of rigid docking with ionic/charged Cys and His in the active site (Figure 3A) in order to evaluate the compounds studied in this work, with the M^pro^ and PL^pro^ enzymes at their crucial initial points in the catalytic cycle. In M^pro^, the His41 residue functions as a base, deprotonating Cys145 and thereby enhancing its nucleophilicity. The resulting deprotonated cysteine (thiolate) becomes a highly reactive nucleophile, facilitating the formation of the enzyme–substrate (or enzyme–inhibitor) complex [65,66]. Similarly, in PL^pro^, the His272 residue acts as a base, deprotonating Cys111, which also increases its nucleophilicity. Asp286 interacts with His272 through a hydrogen bond, and this interaction stabilizes the position and orientation of His272, enabling it to function as an efficient catalytic base. The deprotonated cysteine (thiolate) then acts as a nucleophile, enabling the formation of the enzyme–substrate (or enzyme-inhibitor) complex [66,67]. The description and binding poses of the flexible docking simulations and the binding poses of CF and its M1 and M2 metabolites of the rigid docking simulation with the active site Cys and His charged are included in the Supplementary Materials (Figures S1–S8).

Two conformers were analyzed for each performed docking. The conformer with the largest negative energy (Table 1 and Table S1) was used to analyze in which situations there would be a more thermodynamically favorable interaction between the enzymes and the ligands. The other chosen conformer presented the shortest S···S distance (Cys and 1,2,4-thiadiazole moiety) (Table 2 and Table S1). In general terms, the different docking methodologies carried out and analyzed in this work for M^pro^ presented more thermodynamically favorable binding free energies (∆G, kcal·mol^−1^) (Table 1 and Table 2. ^a^M^pro^) (Tables S1 and S2. ^c^M^pro^) when compared to PL^pro^ (Table 1 and Table 2. ^b^PL^pro^) (Tables S1 and S2. ^d^PL^pro^). This behavior has already been observed in similar *in silico* studies using these proteases [14,19,42]

#### 3.1.1. M^pro^ Docking Simulations

**Figure 4:**
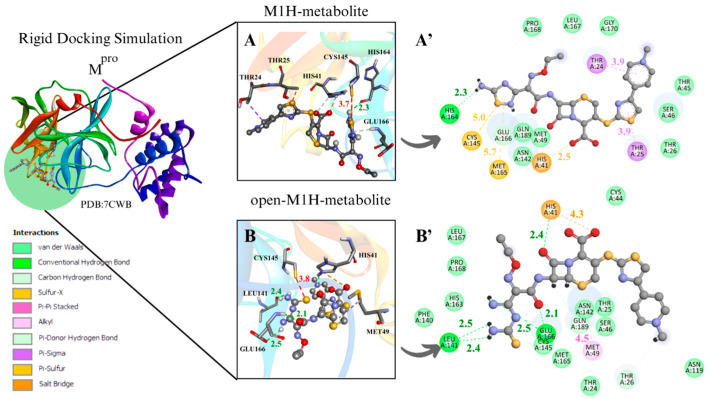

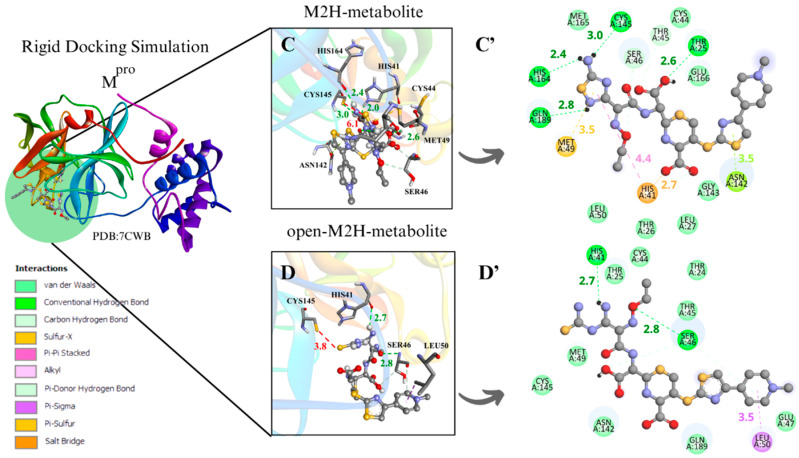
Rigid docking focused on active site with Cys and His charged in most favorable S···S interaction conformer. M^pro^ result compilation of 3D and 2D diagrams, with interaction distances between active site environment and ligands. (**A**) M1H metabolite 3D scheme, ∆G = −7.8 kcal·mol^−1^. (**A’**) M1H metabolite 2D scheme. (**B**) Open-M1H metabolite 3D, ∆G = −7.4 kcam·mol^−1^. (**B’**) Open-M1H metabolite 2D scheme. (**C**) M2H metabolite 3D scheme, ∆G = −7.5 kcal·mol^−1^. (**C’**) M2H metabolite 2D scheme. (**D**) Open-M2H metabolite 3D, ∆G = −6.9 kcal·mol^−1^. (**D’**) Open-M2H metabolite 2D scheme. Legend of intermolecular interactions is shown in bottom left corner. Distances are in Å. In order to optimize visualization of interaction of ligand with main amino acids of active site, some amino acids in environment are only highlighted in 2D visualization.

To evaluate the M^pro^ enzyme at the initial and critical stage of its catalytic cycle, along with the metabolites at a strategic point in the proposed inhibitory mechanism of cysteine enzymes by 1,2,4-thiadiazoles for enzyme–ligand complex formation, the conformers with the most favorable interaction distance between the Cys145 sulfur atom (S···S) and the 1,2,4-thiadiazole group of the metabolites M1H, open-M1H, M2H, and open-M2H were analyzed within the rigid molecular docking simulation focused on the active site containing charged Cys and His residues (Figure 4).

The binding poses obtained from the different docking analyses in the M^pro^ active site disclosed that the sulfur atom of the 1,2,4-thiadiazole heterocycle of the antibiotic CF and its metabolites interacted with the thiolate group of Cys145 and with the surrounding residues. When measuring the distances of Cys145 S···S 1,2,4-thiadiazole in the conformer characterized by the largest negative ∆G binding energy, the rigid simulation focused on the active site with Cys and His charged demonstrated more favorable S···S distances for the M1 metabolite (4.3 Å), M1H metabolite (4.2 Å), and open-M1H metabolite (4.5 Å); the binding free energies in these analyzed conformers of M^pro^ presented values from −8.5 to −7.5 (∆G, kcal·mol^−1^) (Table 1. ^a^M^pro^).

Regarding the analyses of the conformer with the most favorable S···S interaction distances between Cys145 and 1,2,4-thiadiazole, the rigid simulation focused on the active site with Cys and His charged, the M2 metabolite presented a distance of 3.5 Å, and the binding free energies in these analyzed conformers of M^pro^ presented values from −8.5 to −6.9 (∆G, kcal·mol^−1^) (Table 2. ^a^M^pro^).

In the analysis of the rigid simulation focusing on the active site with charged Cys and His, different behaviors were observed for the interactions of metabolites M1 and M2. While the S···S interaction distances between the M1 metabolite, the M1H metabolite, and the open-M1 metabolite structures were similar (3.7–3.8 Å) (Figure S1B’) (Figure 4A,B), the S···S distances between the M2 metabolite, the M2H metabolite, and the open-M2H metabolite varied from 3.5 to 6.1 Å (Figure S1E’) (Figure 4C,D). There were also variations in ∆G. While the M1H metabolite and open-M1H metabolite presented thermodynamic values of −7.8 and −7.4 kcal·mol^−1^, respectively, the M2H metabolite and open-M2H metabolite presented ∆G values of −7.5 and −6.9 kcal·mol⁻^1^, respectively, suggesting a variation in thermodynamic energy and stability between the structures of M2H and open-M2H metabolites (Figure 4) (Table 2. ^a^M^pro^).

Although M1H and open-M1H metabolites showed similar interaction distances between the thiolate of Cys145 and the sulfur of the 1,2,4-thiadiazole heterocycle, the surrounding amino acid environment is quite different (Figure 4A,B). The M1H metabolite exhibits a π-sulfur interaction between the sulfur of Met49 and Cys145 and the 1,2,4-thiadiazole heterocycle, together with π-sigma interactions in Thr24 and Thr25 and one H-bond with His164, as shown the 2D scheme (Figure 4A’). The open-M1H metabolite, the metabolite formed after the opening of the 1,2,4-thiadiazole heterocycle, is characterized by the presence of a larger number of hydrogen bonds in the active site environment and an increase in Van Der Waals interactions, as shown in the 2D scheme (Figure 4B’).

In the analysis of the interactions between the M2H metabolite and the open-M2H metabolite with M^pro^, in addition to the difference in the S···S binding poses, there is also a difference in the surrounding amino acid environment. In the M2H metabolite, there is a greater number of H-bonds with His164, Cys145, Thr25, and Gln189, along with π-sulfur interactions with Met49, as shown in the 2D scheme, compared to those observed with the open-M2H metabolite (Figure 4C’). The open-M2H metabolite has two H-bonds with His41 and Ser46, and some Van der Waals interactions (Figure 4D,D’). This suggests that although the Cys145 S···S 1,2,4-thiadiazole interaction is not the most favorable (6.1Å), there are more interactions involving the M2H metabolite, and thermodynamically this conformer presents better ∆G = −7.5 kcal·mol^−1^, compared with the open-M2H metabolite that presents ∆G = −6.9 kcal·mol^−1^ (Table 2. ^a^M^pro^). This could confer to the M2H metabolite greater stability in the M^pro^ active site environment.

Among the metabolites analyzed for the M^pro^ enzyme, in the rigid docking configuration with the active site charged, the M1H metabolite presented more favorable results in relation to its thermodynamics, Cys145 and 1,2,4-thiadiazole moiety (S···S) distances, and interactions in the active site environment. It has ∆G of −8.5 kcal·mol^−1^ and an S···S distance of 4.2 Å in the conformer with the largest negative ∆G (Table 1. ^a^M^pro^), and ∆G of −7.8 kcal·mol^−1^ and an S···S interaction distance of 3.7 Å in the conformer with the most favorable S···S interaction distances (Table 2. ^a^M^pro^) (Figure 4A,B).

In the two molecular docking methodologies used to evaluate M^pro^ with CF, its metabolites, and its derivatives, the presence of amino acids, His41, Ser46, Ans142, Cys145, His163, Glu166, and Gln189, was observed, carrying out diverse intermolecular interactions (Figure 4) (Figures S1–S4). Similar interactions were observed in several M^pro^ inhibitors, both in cases of covalent inhibitors, such as N3, PDB (6LU7) and MPI3, PDB (7JQ0), with interactions between Cys145, His163, Glu166, and Gln189 [27,68,69,70], and in cases of non-covalent inhibitors, such as ML300- PDB (7LME) and ML188- PDB (7L0D), presenting intermolecular interactions between Ser46, His163, and Glu166 [68,71,72].

#### 3.1.2. PL^pro^ Docking Simulations

**Figure 5:**
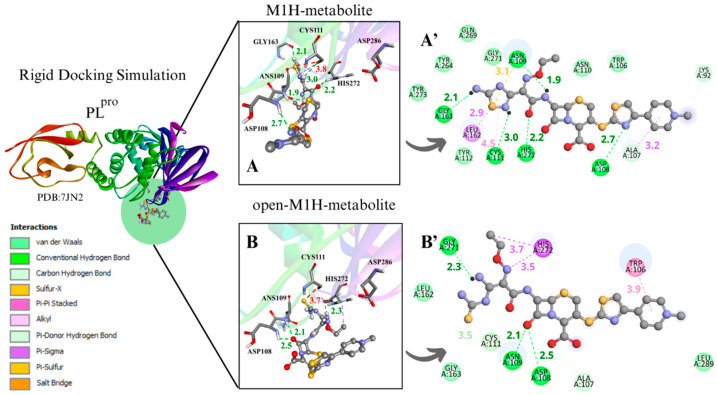

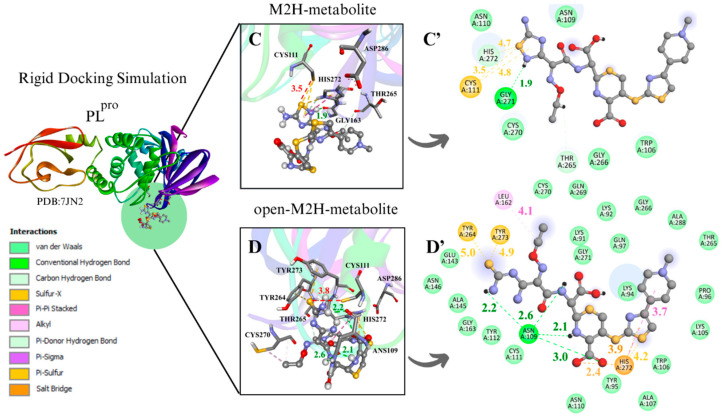
Rigid docking focused in the active site with Cys and His charged in the most favorable S···S interaction conformer. PLpro result compilation of 3D and 2D diagrams, with the interaction distances between the active site environment and the ligands. (**A**) M1H metabolite 3D scheme, ∆G = −5.6. (**A’**) M1H metabolite 2D scheme. (**B**) Open-M1H metabolite 3D, ∆G = −5.8. (**B’**) Open-M1H metabolite 2D scheme. (**C**) M2H metabolite 3D scheme, ∆G = −5.3. (**C’**) M2H metabolite 2D scheme. (**D**) Open-M2H metabolite 3D, ∆G = −5.4. (**D’**) Open-M2H metabolite 2D scheme. The legend of the intermolecular interactions is shown in the bottom left corner. Distances are in Å. In order to optimize the visualization of the interaction of the ligand with the main amino acids of the active site, some amino acids in the environment are only highlighted in the 2D visualization.

To evaluate the PL^pro^ enzyme at the initial and critical stage of its catalytic cycle, along with the metabolites at a strategic point in the proposed inhibitory mechanism of cysteine enzymes by 1,2,4-thiadiazoles for enzyme–ligand complex formation, the conformers with the most favorable interaction distance between the Cys111 sulfur atom (S···S) and the 1,2,4-thiadiazole group of the metabolites M1H, open-M1H, M2H, and open-M2H were analyzed within the rigidly focused active site containing charged Cys and His residues (Figure 5).

The binding poses obtained in the rigid and flexible docking analyses in the PL^pro^ active site showed that the sulfur atom of the 1,2,4-thiadiazole heterocycle of the antibiotic CF and its studied metabolites interacted with the thiolate group of Cys111 and nearby residues (Figure 5) (Figures S5–S8). In general, it was observed that the binding free energies were less favorable with PL^pro^ than with M^pro^ (Table 1, Table 2, Tables S1 and S2). This was already observed in previous studies with CF and other chalcogen compounds [14,19,42,73].

Analyzing the distances between Cys111 and the 1,2,4-thiadiazole moiety (S···S) in the most favorable negative ∆G conformer for the rigid simulation focused on the active site with charged Cys and His demonstrated more favorable S···S distances for the open-M2H metabolite (3.9 Å) with ∆G = −5.8 kcal·mol^−1^ (Figure S6G*) (Table 1. ^b^PL^pro^).

The analysis of the results of the conformer with the most favorable S···S interaction distances between the Cys111 S···S and the 1,2,4-thiadiazole moiety demonstrated that in the rigid simulation focused on the active site with charged Cys and His, there are favorable distances to the open-M1H metabolite, with distances of 3.7 Å and a binding free energies value −5.8 kcal·mol^−1^, and to the M2H metabolite, with a distance of 3.5 Å and a binding free energies value of −5.3 kcal·mol^−1^ (Figure 5B,C) (Table 2. ^b^PL^pro^).

The structures derived from metabolite M1, specifically the conformers with the most favorable S···S interaction distances between the sulfur atom of Cys111 and the 1,2,4-thiadiazole moiety, exhibited binding free energies ranging from −6.4 to −5.2 kcal·mol⁻^1^. The corresponding S···S distances were within 3.7–3.8 Å (Table 2. ^b^PL^pro^) (Figure 5A,B) (Figure S5B*). The metabolites M1H and open-M1H demonstrated similar interaction distances, as well as similarities in the types of chemical interactions and the surrounding amino acid environment (Figure 5A,B). In contrast, structures derived from the M2 metabolite showed a wider range of S···S distances, spanning from 6.5 Å to 3.5 Å (Figure 5C,D) (Figure S5E*).

For instance, the M1H metabolite presents H-bonds with the residues Ans 109, Cys111, Asp 108, Gly163, and His272, as well as sulfur–X (X = O, N, S) interactions [74] between the sulfur of the heterocycle 1,2,4-thiadiazole and the carbonyl oxygen of the peptide bond of Ans109, together with π-sigma on Leu162, as demonstrated in the 2D scheme (Figure 5A’). The open-M1H metabolite, on the other hand, presents less H-bonds, compared with the M1H metabolite, between the amino acids Ans109, Asp108, and Gly271, as well as π-sigma interactions with one amino acid of the active site, His272 (Figure 5B’).

The M2H and open-M2H metabolite results in the conformer with the most favorable interaction distance between the Cys111 S···S and 1,2,4-thiadiazole reveal some variations in the intermolecular interactions with the environment active site. The M2H metabolite presents π-sulfur-type interactions with Cys111 and His272, one sulfur–X (X = N, O, S) [74] interaction between the sulfur of the heterocycle 1,2,4-thiadiazole and the sulfur of Cys111, H-bonds with Gly271, and some Van der Waals interactions (Figure 5A’,B’). The open-M2H metabolite in the conformer with the most favorable interaction distance between the Cys111 S···S and 1,2,4-thiadiazole presents π-sulfur interactions between amino acids Tyr263 and Tyr264 and the sulfur of the 1,2,4-thiadiazole heterocycle, as well as H-bonds with Ans109 and His272; in contrast, there are a greater number of Van der Waals interactions compared with the M2H metabolite, as shown in the 2D schemes (Figure 5C’,D’).

Between the metabolites analyzed for the PL^pro^ enzyme, in the rigid docking configuration with the active site charged, the open-M2H metabolite presents more favorable results in relation to its thermodynamics, Cys111 and 1,2,4-thiadiazole moiety (S···S) distances, and interactions in the active site environment. It has ∆G of −5.8 kcal·mol^−1^ and an S···S distance of 3.9 Å in the conformer with the largest negative ∆G (Table 1. ^d^PL^pro^) and ∆G of −5.4 kcal·mol^−1^ and an S···S interaction distance of 3.8 Å in the conformer with the most favorable S···S interaction distances (Table 2. ^b^PL^pro^) (Figure 5D,D’).

The presence of amino acids, such as Trp106, Asp108, Ans109, Cys111, Gly163, Glu166, Gly271, His272, and Asp286, was observed, carrying out diverse intermolecular interactions in all molecular docking methodologies used to evaluate PL^pro^ with CF, its metabolites, and its derivatives (Figure 5) (Figures S5–S8). Similar interactions were observed in PL^pro^ inhibitors, both in cases of covalent inhibitors, such as 252, PDB (8EVA) and 358, PDB (8IHO), with interactions between Trp106, Ans 109, Cys111, and His272 [67,75,76], and in cases of non-covalent inhibitors, such as Jun11313- PDB (8UVM), presenting interactions between Trp106, Ans109, and Gly163 [77].

The use of different molecular coupling methodologies for the same target increases reliability, reducing the tendency for isolated results. This approach allows for a more complete exploration of the binding environment, improving the identification of binding modes and affinity hotspots. Cross-validation of results between methods increases confidence in enzyme–ligand interactions, assisting in the selection of candidates for experimental validation and offering more assertive insights into complex biological targets [78,79,80].

In order to understand how the metabolites analyzed above could behave as targets in the active site of SARS-CoV-2 proteases, molecular dynamics simulations were carried out, and these will be discussed in the following.

### 3.2. Molecular Dynamics Simulations

In both enzymes, the thermodynamically most favorable conformer of the rigid docking simulations focused in the active site with Cys and His charged was utilized (Table 1. ^a^M^pro^–^d^PL^pro^) in order to evaluate the M1H, M2H, open-M1H, and open-M2H metabolites with the M^pro^ and PL^pro^ enzymes at their crucial points in the catalytic cycle, enabling the formation of the enzyme–substrate (or enzyme–inhibitor) complex. After running 100 ns productions (see the computational details), we compared the overall root mean square deviation (RMSD) of the backbone atoms of the four metabolites in the complex with M^pro^ and PL^pro^.

**Figure 6:**
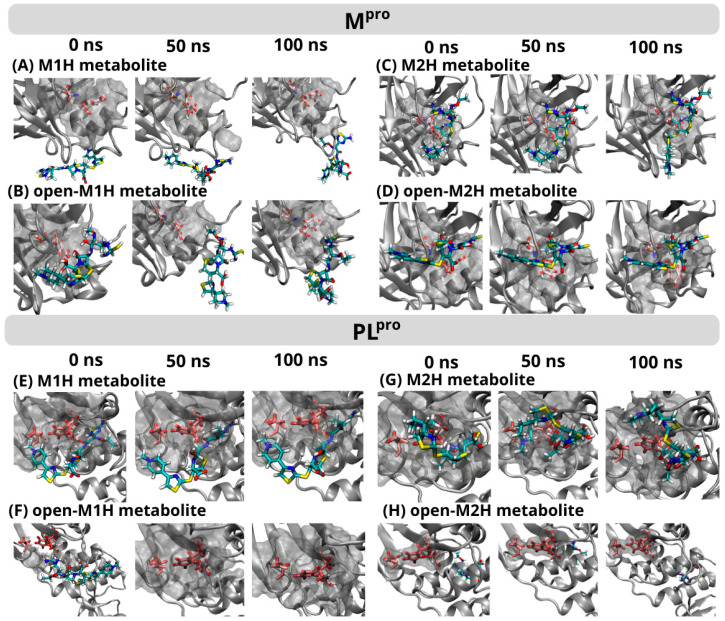
Frame-to-frame evolution of the trajectories for each of the four metabolites analyzed in the conformational dynamics for M^pro^ (**top**) and PL^pro^ (**below**) within the protein’s binding pocket over a 0 ns, 50 ns, and 100 ns production time. The simulation area for each protein is represented by a gray cloud, with only the catalytic dyad and triad in each enzyme’s binding pocket highlighted (Mpro Cys145, His41/PLpro Cys111, His272, Asp286) using a ball-and-stick representation. The metabolites M1H, M2H, open-M1H, and open-M2H are depicted as colorful sticks. The complete timeframe figures are available in the SI (Figures S13 and S14).

**Figure 7:**
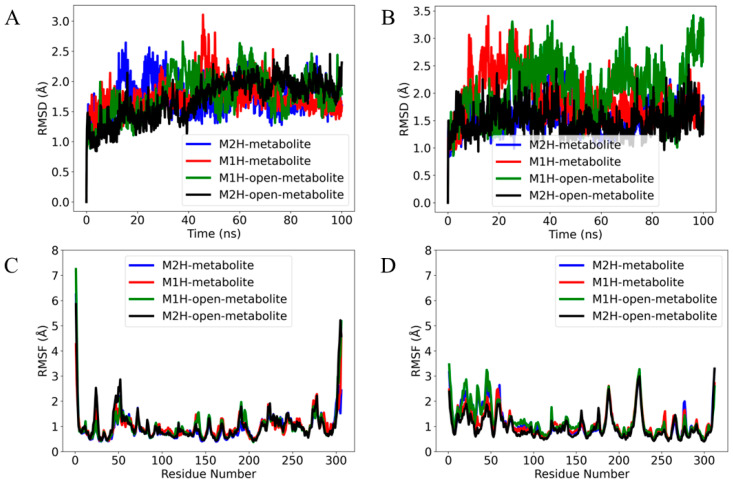
The root mean square deviation (RMSD) of the α (alpha) carbon atoms of the complexes in (**A**) M^pro^ and (**B**) PL^pro^ over a 100 ns production time. The RMSD values are calculated with respect to each metabolite’s conformation at 0 ns production time. The root mean square fluctuation (RMSF) of (**C**) M^pro^ and (**D**) PL^pro^ residues in response to binding different metabolite states over a 100 ns molecular dynamics simulation. The plot displays the evolution of the RMSD and RMSF for four metabolite complexes: M2H (blue), M1H (red), open-M1H (green), and open-M2H (black).

The RMSD plot for the M^pro^ ligands provides a visual representation of ligand stability and conformational dynamics within the protein’s binding pocket (Figure 6). Each colored trace corresponds to one of the four different metabolite states: M1H, M2H, open-M1H, and open-M2H (Figure 7). After the initial equilibration, the RMSD values for each metabolite reach a plateau, indicating that each ligand reaches a relatively stable conformation within the binding site of M^pro^ and PL^pro^. However, the degree of stability differs among the metabolites.

The M2H metabolite and the open-M2H metabolite complexes (as shown by the blue and black traces, respectively) (Figure 7A) display less fluctuation in their RMSD values, suggesting a more stable interaction within their respective binding pockets. In contrast, the M1H metabolite and the open-M1H metabolite (red and green traces) show higher and more frequent peaks, indicating slightly more dynamic behavior and flexibility in their binding conformations. No significant long-term upward or downward trends are observed, suggesting that none of the metabolites is undergoing a slow process of dissociation or significant conformational drift over the time scale of the simulation. The RMSD analysis of the metabolites within the PL^pro^ binding site (Figure 7B) reveals distinct patterns of conformational stability across the four metabolite states over the course of the simulation. Compared to their behavior in the M^pro^ complexes (Figure 7A), the metabolites exhibit a notably different dynamic profile in PL^pro^. The M2H and open-M2H metabolites maintain a lower RMSD, suggesting stable binding throughout the simulation timeframe, similar to their behavior in M^pro^. Conversely, the M1H and open-M1H metabolites show greater variability in their RMSD trajectories when bound to PL^pro^, implying a less stable interaction than with M^pro^. The observed higher peaks and broader fluctuations in RMSD suggest that these metabolites within the complexes may adopt multiple conformations within the PL^pro^ binding site or engage in more dynamic interactions with the protein.

At the initial 0 ns time point, the M1H metabolite formed interactions with a specific set of amino acids within the PL^pro^ binding site at 3.5 Å, including Trp103, Ala104, Asp105, Asn106, Gln266, Cys108, Tyr109, Cys267, Gly268, His269, Tyr270, Asp283, Leu286, Leu159, and Gly160 (Figure 6). These interactions were largely maintained at the 50 ns mark, with the exception of Asp283 and Cys267, indicating a dynamic interaction landscape. At the 100 ns time point, the interaction profile reverted to closely resemble that observed at 0 ns, with the re-establishment of interactions with Asp283 and Cys267. In contrast, the M2H metabolite demonstrated an inverse interaction pattern. It showed a stable interaction with the M^pro^ active site, contrasting with the unstable interaction observed for the M1H metabolite (Figure 6). However, the interaction of the M2H metabolite with the PL^pro^ active site was unstable, mirroring the M1H interaction with M^pro^. Furthermore, the open-M1H and open-M2H derivatives of the metabolites exhibited generally unstable interactions with both M^pro^ and PL^pro^, indicating a significant impact of the metabolite’s structural conformations on its binding stability.

The root mean square fluctuation (RMSF) analysis of M^pro^ in the presence of different metabolites reveals insights into the dynamic nature of protein–ligand interactions. In the core of the protein, Met49, Asn72, and Met165 exhibit notable fluctuations, which could be indicative of their involvement in the protease’s functional dynamics, potentially affecting substrate recognition and catalysis. Comparing the RMSF profiles across the different metabolites provides a differential view of how each metabolite affects the protein’s dynamics. The open-M1H and open-M2H metabolites (green line) induce fluctuations of about 1 Å in certain residues compared to the inactive state (blue line); this could suggest a slight allosteric modulation of the protein structure, which might alter its activity or ligand affinity.

The total number of fluctuations across the PL^pro^ residues is less than four, indicating a more stable system. The RMSF profile shows that the metabolite states modulate the flexibility of residues within the thumb (Asp164) and the palm (Tyr269) domains, known to be essential for broad-spectrum antiviral drug design. The principal component analysis (PCA) was performed to reduce the dimensionality of the molecular dynamics simulation data and to identify the major patterns of motion within the protein structures of M^pro^ and PL^pro^. The analysis was conducted on the backbone atoms to capture the overall protein dynamics, post-alignment to the initial simulation frame. Figure S13 illustrates the PCA results for the main protease complexes. The active and inactive state display a more dispersed distribution along both PC1 and PC2, indicative of higher conformational variability. Conversely, the open-M1H and open-M2H metabolite states formed more compact clusters, suggesting a more restrained conformational landscape. In Figure S13, showing the PCA for the M^pro^ and PL^pro^ complexes, the substantial overlap in the distribution of points across all metabolic states indicates that these complexes maintain similar conformational profiles throughout the simulations. This suggests that the M^pro^ and PL^pro^ complexes, irrespective of their metabolic states, display a consistent structural integrity over the duration of the trajectories examined.

### 3.3. M^pro^ and PL^pro^ Enzyme Inhibition Analysis

**Figure 8:**
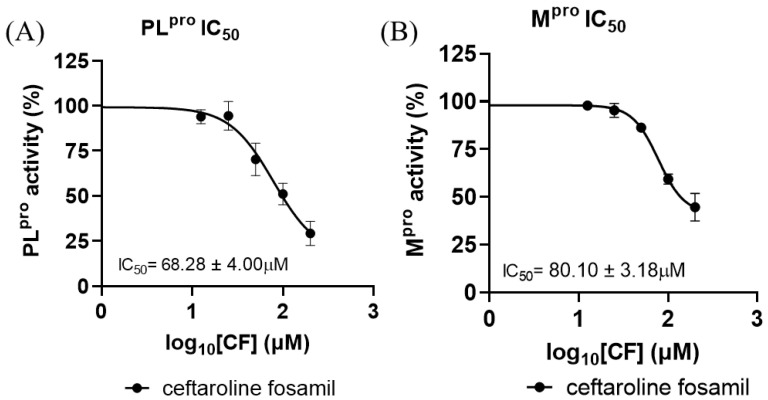
Dose–response curves for CF tested against SARS-CoV-2 (**A**) PL^pro^ and (**B**) M^pro^ recombinant enzyme analysis. All experiments were performed in triplicate, and the data are expressed as the mean ± standard deviation.

To substantiate the *in silico* results, the inhibitory potency of CF was tested with SARS-CoV-2 proteases (Figure 8). CF inhibited PL^pro^ with an IC_50_ of 68.28 ± 4.00 μM and M^pro^ with an IC_50_ of 80.10 ± 3.18 μM (Figure 8A,B). According to the results obtained in this work, the enzyme with the best inhibition rate is PL^pro^. This result corroborates findings previously reported by Delgado et al. in a molecular dynamics study, where it was verified that there are greater interactions and shorter distances between the 1,2,4-thiadiazole group of M1 and the Cys of the active site of PLpro [73]. However, the inhibitory potency of CF was higher than that reported from *in vitro* tests against the M^pro^ and PL^pro^ enzyme [81,82,83]; ebselen, for example, inhibits M^pro^ with an IC_50_ of 0.67 μM and PL^pro^ with an IC_50_ of 2.35 μM, while disulfiram inhibits M^pro^ and PL^pro^ with an IC_50_ of 2.45 μM and 13.06 μM, respectively [84,85]. Nirmatrelvir, one of the combined protease inhibitors used for Paxlovid, approved for COVID-19, inhibits M^pro^ with an IC_50_ of 19.2 nM [80,86,87].

Indeed, the IC_50_ values found for the two viral proteases were relatively high but not so distant from the concentrations of CF and M1 found in human blood plasma, at 42.5 µM (in the first hour of 600 mg across a 12 h dosing regimen) [44]. However, the values found for the analyzed viral proteases and the concentrations in human epithelial lung fluids (12.6 µM—in the first hour of 600 mg q across a 12 h dosing regimen) [44] presented discrepancies.

After the *in vitro* biochemical tests with M^pro^ and PL^pro^, we sought to analyze the potential inhibitory effects of CF against SARS-CoV-2 replication in two cellular models in order to understand whether there would be more promising values in the overall process and not only with the target on SARS-CoV-2 proteases.

### 3.4. In Vitro Analysis of Ceftaroline Fosamil Against SARS-CoV-2 Replication

#### 3.4.1. The Effect of Ceftaroline Fosamil on Calu-3 Cells Viability

**Figure 9:**
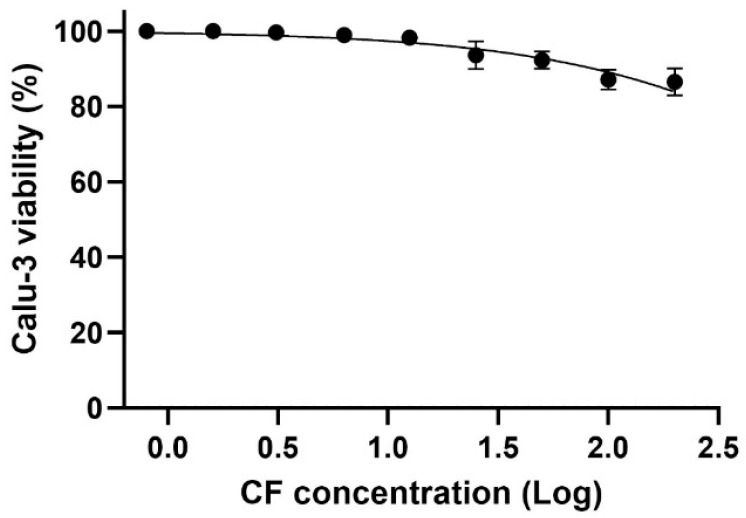
The effect of CF on the Calu-3 cells viability. The Calu-3 cells were exposed to different concentrations (0.8, 1.6, 3.1, 6.3, 12.5, 25, 50, 100, and 200 µM) of the drug for 72 h, at 37 °C and 5% CO_2_. Cell viability was determined using the methylene blue staining procedure (*n* = 3).

For the *in vitro* assays, we utilized a cell model that mimics natural infection in human type II pneumocytes, specifically Calu-3 cells. Due to their physiological and structural characteristics, Calu-3 cells have been widely used in studies of SARS-CoV-2 infection. The data show that even at the highest concentration tested, cell viability remained above 80%, indicating a CC_50_ above 200 µM for CF in the evaluated cell model (Figure 9).

#### 3.4.2. The Effect of Ceftaroline Fosamil on SARS-CoV-2 Replication in Calu-3 Cells

**Figure 10:**
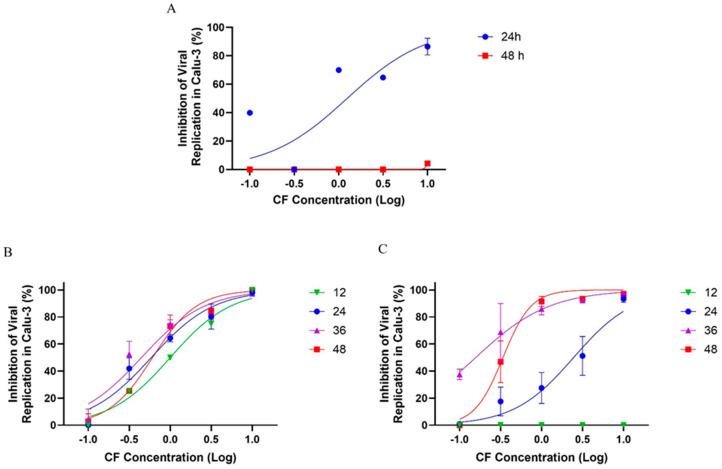
The antiviral effect of CF on SARS-CoV-2 replication in Calu-3 cells. Calu-3 cells infected with an MOI of 0.01 (**A**,**B**) or 0.1 (**C**) and exposed to CF under a semi-log concentration curve (0.1, 0.316, 1, 3.16, and 10 µM) were treated without (**A**) or with (**B**,**C**) pulses of treatment in different times post infection (12, 24, 36, and 48 h). The data represent the results of four independent experiments with four technical replicates. The value of R2 ranged from 0.90 to 0.95 (MOI 0.1) and from 0.92 to 0.98 (MOI 0.01).

First, Calu-3 cells were infected with SARS-CoV-2 at a multiplicity of infection (MOI) of 0.01 and exposed to varying concentrations of CF. After 24 and 48 h post infection (hpi), the supernatants were collected for virus titration. We observed that CF inhibited viral growth as early as 24 hpi, with an EC_50_ of 1.25 µM (Figure 10A). However, after 48 h, the inhibitory effect was not evident, suggesting that CF could have been metabolized to inactive intermediates after two days. To investigate the potential inactivation of CF prolonged incubation, we infected Calu-3 cells with an MOI of 0.01 and 0.1 and treated them with CF for 48 h, but administering new pulses of CF every 12 h after the initial infection.

In the infection model with an MOI 0.01, we observed that the levels of viral replication inhibition of SARS-CoV-2 were dose-dependent, reaching ≥70% inhibition after treatment at a 3.16 µM concentration, independent of the time proposed in this analysis (Figure 10B). Almost 100% of viral inhibition was observed at a 10 µM concentration, and at 1 µM, the viral inhibition was ≥50% after 12, 24, 36, and 48 h of incubation (Figure 10B). In the infection model with an MOI of 0.1, there was no antiviral activity at 12 h of treatment; however, with 24 h of treatment the levels of viral replication inhibition were ≥40% at 3.16 µM and ≥90% at a 10 µM concentration after 24 h of treatment. After 36 and 48 h of treatment with CF, the inhibitory potency of CF increased and the antiviral activity increased to about 80% at 1 µM and to near 100% at 3.16 and 10 µM (Figure 10C). These results with an MOI of 0.01 and 0.1 indicate a potent antiviral effect of CF after treatment with pulses at every 12 h (Figure 10A). Accordingly, the EC_50_ average values for CF in Calu-3 previously infected with an MOI of 0.01 and 0.1 were 0.65 ± 0.24 and 0.24 ± 0.13 µM, respectively (Table 3).

This study investigated the binding and inhibitory potential of the antibiotic CF and its metabolites against SARS-CoV-2 proteases, M^pro^ and PL^pro^, using docking simulations (one rigid and one semi-flexible docking method) focusing on the sulfur atom within the 1,2,4-thiadiazole group for its interactions with cysteine protease. The rigid docking focusing on the active site with Cys and His charged showed that M1H and open-M1H metabolites achieved optimal S···S interactions with M^pro^ Cys145 with distances between the S atoms ranging from 3.7 to 3.8Å (Figure 4). With PL^pro^, rigid docking focusing on the active site with Cys and His charged showed that M2H and open-M2H metabolites presented shorter S···S interactions with Cys111. In both proteases, the analysis exhibited π-sulfur, H-bond, and Van der Walls interactions, while PL^pro^ in M1H and M2H metabolites presented π-sulfur, H-bond, and Van der Walls interactions. In addition, the simulations also indicated sulfur–X interactions (X = N, O, S), specifically an intermolecular interaction between the sulfur of the 1,2,4-thiadiazole heterocycle and the carbonyl oxygen of the peptide bond of asparagine (Ans109) and the sulfur atom of cysteine (Cys111) heteroatoms. The lack of covalent interactions, together with π-sulfur, can confer greater stability between proteins and ligands [74,88,89]. In the molecular dynamics simulations, the M2H metabolite presented more stability with the M^pro^ active site and environment residues, while the PL^pro^ results reveal greater stability with the M1H metabolite and M2H metabolite.

The *in vitro* PL^pro^ inhibition rates were better than those of M^pro^. The CF IC_50_ values increased following pre-incubation with plasma, which simulated M1 metabolite formation. The *in vitro* analysis with Calu-3 cells, a human lung cell model for SARS-CoV-2 infection, showed that CF maintained high cell viability (>80%) even at high concentrations, with a CC_50_ above 200 µM. CF exhibited dose-dependent inhibition of SARS-CoV-2 replication, reaching nearly 100% inhibition at 10 µM under a low MOI (0.01) and around 90% inhibition after 24 h at a higher MOI (0.1) when treated with 10 µM CF. Although it is not possible to extrapolate the findings of the biochemical data to the antiviral data assays, the much higher potency of CF in the antiviral assay may suggest that CF can target additional proteins of SARS-CoV-2, such as structural proteins, Spike, Membrane, Envelope and Nucleocapsid, or accessory proteins (ORF3a, ORF6, ORF7a, ORF7b, ORF8, and ORF10) [66,90,91,92].

These findings open the possibility that CF may exert its antiviral effects through mechanisms beyond the inhibition of M^pro^ and PL^pro^. The observed reduction in viral replication at submicromolar concentrations suggests that CF could interact with other viral or host cellular components essential for the SARS-CoV-2 life cycle. For instance, it is plausible that CF interferes with viral entry, assembly, or budding processes, or modulates host cell pathways critical for viral replication. This hypothesis aligns with evidence from other multimodal antivirals that exhibit broad-spectrum effects by targeting various stages of the viral life cycle [93,94,95]. Therefore, future studies are essential to explore these alternative mechanisms and clarify the full spectrum of CF’s antiviral activity. Understanding these pathways will be pivotal in optimizing CF’s application as a therapeutic agent against SARS-CoV-2 and potentially other viral pathogens.

When compared to other antiviral molecules approved or recommended for emergency use by the FDA, such as Remdesivir, CF exhibits EC_50_ values that fall within a similar range or, in some cases, are even more effective. For instance, Remdesivir, approved for COVID-19 treatment, has an EC_50_ around 1.76 µM in Calu-3 cells, positioning CF as a potentially competitive alternative in terms of antiviral efficacy [96].

Another notable molecule is the monoclonal antibody combination Bamlanivimab, which exhibits strong antiviral effects but with a more complex administration and logistics profile due to the need for precise dosage control and a specific application environment [97,98]. In comparison, the use of CF, which demonstrated high antiviral efficacy with administration every 12 h, could provide a more practical and adaptable approach for treatment across different stages of infection and healthcare settings.

Furthermore, the observation that CF exhibited significant antiviral efficacy even after 48 h in MOI 0.1 infection models demonstrates its resilience and potential for prolonged use, similar to what has been observed with other therapies, such as Paxlovid, a combination protease inhibitor approved for COVID-19. Paxlovid showed a significant reduction in viral load in clinical studies [99,100,101], suggesting that combination therapy can be an effective, though more costly, approach. The CF results in this study indicate a promising path for an accessible and effective antiviral agent with the potential to reduce both viral load and infection duration with a less invasive treatment regimen. Clinical studies are being carried out with CF, such as the Randomized, Embedded, Multifactorial Adaptive Platform Trial for Community-Acquired Pneumonia (REMAP-CAP) [102,103]. Soriano et al. carried out evaluations of patterns and outcomes of treatment with CF in adults with community-acquired pneumonia (CAP); the prodrug of CF was effective in treating CAP, in Europe and Latin America, with a 60% decrease in patients’ ICU stay [104].

## 4. Conclusions

In this study, the possible inhibition of the Cys proteases of SARS-CoV-2, M^pro^ and PL^pro^, was evaluated by using as a repurposed drug the FDA-approved antibiotic CF, its metabolites, and its derived structures, combining *in silico* and *in vitro* analyses. Our results demonstrate that CF and its metabolites are good inhibitors of PL^pro^ and M^pro^. Interestingly, PL^pro^ has been identified as the preferred *in vitro* biochemical target and as the *in silico* target in molecular dynamics for the M1H and M2H metabolites, while M^pro^ is the preferred *in silico* target in molecular dynamics for the M2H metabolite. The docking analyses presented better thermodynamic results for M^pro^ and the analyzed metabolites, while the docking results for PL^pro^ present more intermolecular interactions between the active site environment. Additionally, CF demonstrated the ability to inhibit viral replication in the Calu-3 cell model at submicromolar concentrations when administered at 12 h intervals. These results offer significant insights for the development of novel COVID-19 therapeutics by leveraging the reactivity of the sulfur atom in the 1,2,4-thiadiazole moiety. However, when comparing the results of enzyme inhibition with the viral replication inhibition data, it becomes evident that Cys proteases are probably not the main pathways of the antiviral CF effect. The greater potency of CF in cellular assays suggests that it may interact with other viral proteins, such as structural or accessory proteins, or even host cellular pathways involved in the viral life cycle. This observation highlights an important aspect of drug discovery: while theoretical and *in silico* approaches provide valuable initial insights, experimental validation is essential to accurately characterize the mechanisms of action of repurposed compounds. The discrepancies observed in this study emphasize that theoretical hypotheses must be corroborated with empirical data to ensure the accurate interpretation of a molecule’s biological activity. Although there are studies on the use of this antibiotic in patients with COVID-19 [45], greater correlations between treatment and survival have not been analyzed. Therefore, future investigations should aim to elucidate the alternative mechanisms by which CF inhibits viral replication, exploring its potential interactions with other viral or host targets. Such studies will be crucial to better understand CF’s full antiviral potential and to guide its clinical application as a multimodal therapeutic agent. Based on the results of this work, we believe that further evaluations should be carried out.

## Figures and Tables

**Table 1:** Predicted binding free energies (∆G, kcal·mol^−1^) between M^pro^ and PL^pro^ and rigid binding site for CF and metabolites with conformer presenting largest negative ∆G.

	^a^ M^pro^		^b^ PL^pro^	
Molecule	∆G	dist. (Å) S^−^*∙∙∙S	∆G	dist. (Å) S^−^*∙∙∙S
		(Cys 145)		(Cys 111)
ceftaroline fosamil	−8.5	8.3	−5.9	8.8
M1 metabolite	−8.5	4.3	−6.4	12.5
M1H metabolite	−8.5	4.2	−6.4	7.6
open- M1H metabolite	−7.9	4.5	−6.3	6.2
M2 metabolite	−7.8	5.5	−5.8	9.0
M2H metabolite	−7.6	7.9	−6.0	8.3
open-M2H metabolite	−7.5	5.7	−5.8	3.9

a—M^pro^ rigid docking focusing on the active site with Cys and His charged, e—semi-flexible docking with Cys and His charged, b—PL^pro^ rigid docking focusing on the active site with Cys and His charged. S^−^*∙∙∙S indicates the distance (in Å) of the sulfur atom of thiolate of Cys145 and Cys111 to the sulfur atom of the 1,2,4-thiadiazole heterocycle. The color gradient from green to red represents the favorability of the interaction: the greener the color, the more favorable the interaction; as the gradient shifts to yellow, the interaction becomes intermediate; and as it transitions to red, it indicates less favorable interactions.

**Table 2:** Predicted binding free energies (∆G, kcal·mol^−1^) between M^pro^ and PL^pro^ and rigid binding site for CF and metabolites with conformer presenting most favorable S···S interaction distances.

	^a^ M^pro^		^b^ PL^pro^	
Molecule	∆G	dist. (Å) S^−^*∙∙∙S	∆G	dist. (Å) S^−^*∙∙∙S
		(Cys 145)		(Cys 111)
ceftaroline fosamil	−8.5	4.2	−5.4	3.9
M1 metabolite	−8.0	3.7	−6.4	3.8
M1H metabolite	−7.8	3.7	−5.6	3.8
open- M1H metabolite	−7.4	3.8	−5.8	3.7
M2 metabolite	−7.6	3.5	−5.2	6.5
M2H metabolite	−7.5	6.1	−5.3	3.5
open-M2H metabolite	−6.9	3.8	−5.4	3.8

a—M^pro^ rigid docking focusing on the active site with Cys and His charged, e—zemi-flexible docking with Cys and His charged, b—PL^pro^ rigid docking focusing on the active site with Cys and His charged. S^−^*∙∙∙S indicates the distance (in Å) of the sulfur atom of thiolate of Cys145 and Cys111 to the sulfur atom of the 1,2,4-thiadiazole heterocycle. The color gradient from green to red represents the favorability of the interaction: the greener the color, the more favorable the interaction; as the gradient shifts to yellow, the interaction becomes intermediate; and as it transitions to red, it indicates less favorable interactions.

**Table 3:** CC_50_ (µM), EC_50_ (µM), and SI for CF in Calu-3 cells.

		12 hpi			24 hpi			36 hpi			48 hpi		
MOI 0.01	CC_50_	EC_50_	SI	R^2^	EC_50_	SI	R^2^	EC_50_	SI	R^2^	EC_50_	SI	R^2^
CF	≥200	0.99 ± 0.09	202.02	0.98	0.58 ± 0.08	344.83	0.95	0.44 ± 0.06	454.54	0.92	0.60 ± 0.05	333.33	0.98
MOI 0.01	CC_50_	EC_50_	SI	R^2^	EC_50_	SI	R^2^	EC_50_	SI	R^2^	EC_50_	SI	R^2^
CF	≥200	NC	NC	NC	2.45 ± 0.35	81.63	0.90	0.15 ± 0.02	1333.33	0.90	0.33 ± 0.02	606.06	0.95

CC_50_: the drug concentration required to promote the death of 50% of treated and non-infected cells. EC_50_: the drug concentration capable of reducing 50% of the maximal inhibitory effect. SI: the selectivity index calculated by the ratio of CC_50_ and EC_50_ values. NC: not calculated. CF: Ceftaroline.

## Data Availability

The original contributions presented in this study are included in the article/supplementary material. Further inquiries can be directed to the corresponding author(s).

## Supplementary Materials

The following supporting information can be downloaded at https://www.mdpi.com/article/10.3390/v17040491/s1: Figure S1: Rigid docking focusing in the active site with Cys and His charged- M^pro^ with ceftaroline fosamil and its metabolites in the favorable S···S interaction distances. (A) ceftaroline fosamil. (B) M1-metabolite. (C) M1H-metabolite. (D) open-M1H-metabolite. (E) M2-metabolite. (F) M2H-metabolite. (G) open-M2H-metabolite. Distances are shown in Å; Figure S2: Rigid docking focusing in the active site with Cys and His charged- M^pro^ with ceftaroline fosamil and its metabolites in the largest negative ΔG binding energy. (A) ceftaroline fosamil. (B) M1-metabolite. (C) M1H-metabolite. (D) open-M1H-metabolite. (E) M2-metabolite. (F) M2H-metabolite. (G) open-M2H-metabolite. Distances are shown in Å; Figure S3: Semi-flexible docking- M^pro^ with ceftaroline fosamil and its metabolites in the favorable S···S interaction distances. (A) ceftaroline fosamil. (B) M1-metabolite. (C) M1H-metabolite. (D) open-M1H-metabolite. (E) M2-metabolite. (F) M2H-metabolite. (G) open-M2H-metabolite. Distances are shown in Å; Figure S4: Semi-flexible docking- M^pro^ with ceftaroline fosamil and its metabolites in the largest negative ΔG binding energy. (A) ceftaroline fosamil. (B) M1-metabolite. (C) M1H-metabolite. (D) open-M1H-metabolite. (E) M2-metabolite. (F) M2H-metabolite. (G) open-M2H-metabolite. Distances are shown in Å; Figure S5: Rigid docking focusing in the active site with Cys and His charged- PL^pro^ with ceftaroline fosamil and its metabolites in the favorable S···S interaction distances. (A) ceftaroline fosamil. (B) M1-metabolite. (C) M1H-metabolite. (D) open-M1H-metabolite. (E) M2-metabolite. (F) M2H-metabolite. (G) open-M2H-metabolite. Distances are shown in Å; Figure S6: Rigid docking focusing in the active site with Cys and His charged- PL^pro^ with ceftaroline fosamil and its metabolites in the largest negative ΔG binding energy. (A) ceftaroline fosamil. (B) M1-metabolite. (C) M1H-metabolite. (D) open-M1H-metabolite. (E) M2-metabolite. (F) M2H-metabolite. (G) open-M2H-metabolite. Distances are shown in Å; Figure S7: Semi-flexible docking- PL^pro^ with ceftaroline fosamil and its metabolites in the favorable S···S interaction distances. (A) ceftaroline fosamil. (B) M1-metabolite. (C) M1H-metabolite. (D) open-M1H-metabolite. (E) M2-metabolite. (F) M2H-metabolite. (G) open-M2H-metabolite. Distances are shown in Å; Figure S8: Semi-flexible docking- PL^pro^ with ceftaroline fosamil and its metabolites in the largest negative ΔG binding energy. (A) ceftaroline fosamil. (B) M1-metabolite. (C) M1H-metabolite. (D) open-M1H-metabolite. (E) M2-metabolite. (F) M2H-metabolite. (G) open-M2H-metabolite. Distances are shown in Å; Figure S9: Comparative timeframe evolution of the trajectories for each of the four metabolites analyzed in the conformational dynamics within the protein’s for M^pro^ binding pocket, over 0 ns to 100 ns production time. The simulation area for each protein is represented by a gray cloud, with only the catalytic dyad and triad in each enzyme’s binding pocket highlighted (M^pro^ Cys145, His41) using a ball-and-stick representation. The metabolites M1H, M2H, open-M1H and open-M2H depicted as colorful sticks; Figure S10: Comparative timeframe evolution of the trajectories for each of the four metabolites analyzed in the conformational dynamics within the protein’s for M^pro^ binding pocket, over 0 ns to 100 ns production time. The simulation area for each protein is represented by a gray cloud, with only the catalytic dyad and triad in each enzyme’s binding pocket highlighted (M^pro^ Cys145, His41) using a ball-and-stick representation. The metabolites M1H, M2H, open-M1H and open-M2H depicted as colorful sticks; Figure S11: Comparative timeframe evolution of the trajectories for each of the four metabolites analyzed in the conformational dynamics within the protein’s for PL^pro^ binding pocket, over 0 ns to 100 ns production time. The simulation area for each protein is represented by a gray cloud, with only the catalytic dyad and triad in each enzyme’s binding pocket highlighted (Cys111, His272, Asp286) using a ball-and-stick representation. The metabolites M1H, M2H, open-M1H and open-M2H depicted as colorful sticks; Figure S12: Comparative timeframe evolution of the trajectories for each of the four metabolites analyzed in the conformational dynamics within the protein’s for PL^pro^ binding pocket, over 0 ns to 100 ns production time. The simulation area for each protein is represented by a gray cloud, with only the catalytic dyad and triad in each enzyme’s binding pocket highlighted (Cys111, His272, Asp286) using a ball-and-stick representation. The metabolites M1H, M2H, open-M1H and open-M2H depicted as colorful sticks; Figure S13: Timeframe evolution of the trajectories for each of the four metabolites analyzed in the conformational dynamics within the protein’s for M^pro^ (top) and PL^pro^ (below) binding pocket, over 0 ns to 100 ns production time. The simulation area for each protein is represented by a gray cloud, with only the catalytic dyad and triad in each enzyme’s binding pocket highlighted (M^pro^ Cys145, His41/ PL^pro^ Cys111, His272, Asp286) using a ball-and-stick representation. The metabolites M1H, M2H, open-M1H and open-M2H depicted as colorful sticks; Figure S14: RMSD values are calculated with respect to each metabolite’s conformation at 0ns production time (A) and (B) are the RMSD of the M^pro^ and PL^pro^ metabolites alone in the complexes. (C) M^pro^ and (D) PL^pro^ Principal component analysis (PCA) plot showing the metabolite complex conformations. Color-coded by metabolic state: M2H (blue), M1H (red), open-M1H (green), and open-M2H (black). The spread along the PC1 and PC2 axes indicates variance in conformation; Table S1: Predicted binding free energies (ΔG, kcal·mol^−1^) between M^pro^ and PL^pro^, flexible binding site, with CF and metabolites for the conformer presenting the largest negative ΔG; Table S2: Predicted binding free energies (ΔG, kcal·mol^−1^) between M^pro^ and PL^pro^, flexible binding site, with CF and metabolites with the conformer presenting the most favorable S···S interaction distances.

## References

[1] Jeronimo P.M.C., Aksenen C.F., Duarte I.O., Lins R.D., Miyajima F. (2024). Evolutionary deletions within the SARS-CoV-2 genome as signature trends for virus fitness and adaptation. J. Virol..

[2] Mukherjee R., Dikic I. (2023). Proteases of SARS Coronaviruses. Encycl. Cell Biol..

[3] Buttle D.J., Mort J.S. (2013). Cysteine Proteases. Encyclopedia of Biological Chemistry: Second Edition.

[4] Pišlar A., Mitrović A., Sabotič J., Fonović U.P., Nanut M.P., Jakoš T., Senjor E., Kos J. (2020). The role of cysteine peptidases in coronavirus cell entry and replication: The therapeutic potential of cathepsin inhibitors. PLoS Pathog..

[5] Gorbalenya A.E., Snijder E.J. (1996). Viral cysteine proteinases. Perspect. Drug Discov. Des..

[6] Zhao J., Qiu J., Aryal S., Hackett J.L., Wang J. (2020). The RNA Architecture of the SARS-CoV-2 3′-Untranslated Region. Viruses.

[7] Sharma A., Farouk I.A., Lal S.K., Martinez-Sobrido L., Toral F.A. (2021). COVID-19: A Review on the Novel Coronavirus Disease Evolution, Transmission, Detection, Control and Prevention. Viruses.

[8] Lecaille F., Kaleta J., Brömme D. (2002). Human and parasitic Papain-like cysteine proteases: Their role in physiology and pathology and recent developments in inhibitor design. Chem. Rev..

[9] Anirudhan V., Lee H., Cheng H., Cooper L., Rong L. (2021). Targeting SARS-CoV-2 viral proteases as a therapeutic strategy to treat COVID-19. J. Med. Virol..

[10] Francés-Monerris A., Hognon C., Miclot T., García-Iriepa C., Iriepa I., Terenzi A., Grandemange S., Barone G., Marazzi M., Monari A. (2020). Molecular Basis of SARS-CoV-2 Infection and Rational Design of Potential Antiviral Agents: Modeling and Simulation Approaches. J. Proteome Res..

[11] V’kovski P., Kratzel A., Steiner S., Stalder H., Thiel V. (2020). Coronavirus biology and replication: Implications for SARS-CoV-2. Nat. Rev. Microbiol..

[12] Harapan H., Itoh N., Yufika A., Winardi W., Keam S., Te H., Megawati D., Hayati Z., Wagner A.L., Mudatsir M. (2020). Coronavirus disease 2019 (COVID-19): A literature review. J. Infect. Public Health.

[13] Yan S., Wu G. (2021). Spatial and temporal roles of SARS-CoV PL^pro^—A snapshot. FASEB J..

[14] Nogara P.A., Omage F.B., Bolzan G.R., Delgado C.D., Orian L., Rocha J.B.T. (2022). Reactivity and binding mode of disulfiram, its metabolites, and derivatives in SARS-CoV-2 PLpro: Insights from computational chemistry studies. J. Mol. Model..

[15] He J., Hu L., Huang X., Wang C., Zhang Z., Wang Y., Zhang D., Ye W. (2020). Potential of coronavirus 3C-like protease inhibitors for the development of new anti-SARS-CoV-2 drugs: Insights from structures of protease and inhibitors. Int. J. Antimicrob. Agents.

[16] Nascimento Junior J.A.C., Santos A.M., Quintans-Júnior L.J., Walker C.I.B., Borges L.P., Serafini M.R. (2020). SARS, MERS and SARS-CoV-2 (COVID-19) treatment: A patent review. Expert. Opin. Ther. Pat..

[17] Rathnayake A.D., Zheng J., Kim K.D., Perera K.D., Mackin S., Meyerholz D.K., Kashipathy M.M., Battaile K.P., Lovell S., Perlman S. (2020). 3C-like protease inhibitors block coronavirus replication in vitro and improve survival in MERS-CoV–infected mice. Sci. Transl. Med..

[18] Lobo-Galo N., Terrazas-López M., Martinez-Martinez A., Diaz-Sanchez A.G. (2020). FDA-approved thiol-reacting drugs that potentially bind into the SARS-CoV-2 main protease, essential for viral replication. J. Biomol. Struct. Dyn..

[19] Nogara P.A., Omage F.B., Bolzan G.R., Delgado C.D., Aschner M., Orian L., Rocha J.B.T. (2021). In silico Studies on the Interaction between Mpro and PLpro from SARS-CoV-2 and Ebselen, its Metabolites and Derivatives. Mol. Inform..

[20] Nogara P.A., Madabeni A., Rocha J.B., Orian L., Emanuelli T., Chitolina M.R., Gasperini A.M., Fonseca D.R. (2022). SARS-CoV-2 enzymes as a drug target: In silico strategies for drug repurposing and drug re-design. International Webinar One Health Over Borders.

[21] Rieder G.S., Nogara P.A., Omage F.O., Duarte T., Corte C.L.D., Rocha J.B.T. (2023). Computational analysis of the interactions between Ebselen and derivatives with the active site of the main protease from SARS-CoV-2. Comput. Biol. Chem..

[22] Pauletto P., Bortili M., Omage F.O., Delgado C.D., Nogara P.A., Orian L., Rocha J.B.T. (2023). In silico analysis of the antidepressant fluoxetine and similar drugs as inhibitors of the human protein acid sphingomyelinase: A related SARS-CoV-2 inhibition pathway. J. Biomol. Struct. Dyn..

[23] Gentile D., Chiummiento L., Santarsiere A., Funcello M., Lupattelli P., Rescifina A., Venut A., Piperno A., Sciortino M.T., Pennisi R. (2024). Targeting Viral and Cellular Cysteine Proteases for Treatment of New Variants of SARS-CoV-2. Viruses.

[24] Isgrò C., Sardanelli A.M., Palese L.L. (2021). Systematic Search for SARS-CoV-2 Main Protease Inhibitors for Drug Repurposing: Ethacrynic Acid as a Potential Drug. Viruses.

[25] Li Q., Kang C.B. (2020). Progress in Developing Inhibitors of SARS-CoV-2 3C-Like Protease. Microorganisms.

[26] Lv Z., Cano K.E., Jia L., Drag M., Huang T.T., Olsen S.K. (2022). Targeting SARS-CoV-2 Proteases for COVID-19 Antiviral Development. Front. Chem..

[27] Jin Z., Du X., Xu Y., Deng Y., Liu M., Zhao Y., Zhang B., Li X., Zhang L., Peng C. (2020). Structure of Mpro from SARS-CoV-2 and discovery of its inhibitors. Nature.

[28] Xiao Y.Q., Long J., Zhang S.S., Zhu Y.Y., Gu S.X. (2024). Non-peptidic inhibitors targeting SARS-CoV-2 main protease: A review. Bioorg. Chem..

[29] Wildner G., Tucci A.R., Prestes A.S., Muller T., Rosa A.S., Borba N.R., Ferreira V.N., Rocha J.B.T., Miranda M.D., Barbosa N.V. (2023). Ebselen and Diphenyl Diselenide Inhibit SARS-CoV-2 Replication at Non-Toxic Concentrations to Human Cell Lines. Vaccines.

[30] Sancineto L., Mangiavacci F., Dabrowska A., Paula-Miszewska A., Obieziurska-Fabisiak M., Scimmi C., Ceccucci V., Kong J., Zhao Y., Ciancaleoni V.N. (2024). New insights in the mechanism of the SARS-CoV-2 Mpro inhibition by benzisoselenazolones and diselenides. Sci. Rep..

[31] Madabeni A., Nogara P.A., Omage F.B., Rocha J.B.T., Orian L. (2021). Mechanistic insight into sars-cov-2 mpro inhibition by organoselenides: The ebselen case study. Appl. Sci..

[32] Omage F.B., Madabeni A., Tucci A.R., Nogara P.A., Bortoli M., Rosa A.S., Ferreira V.N.S., Rocha J.B.T., Miranda M.D., Orian L. (2023). Diphenyl Diselenide and SARS-CoV-2: In silico Exploration of the Mechanisms of Inhibition of Main Protease (Mpro) and Papain-like Protease (PLpro). J. Chem. Inf. Model..

[33] Powers J.C., Asgian J.L., Ekici Ö.D., James K.E. (2002). Irreversible Inhibitors of Serine, Cysteine, and Threonine Proteases. Chem. Rev..

[34] Leung-Toung R., Li W., Tam T., Kaarimian K. (2005). Thiol-Dependent Enzymes and Their Inhibitors: A Review. Curr. Med. Chem..

[35] Leung-Toung R., Wodzinska J., Li W., Lowrie J., Kukrela R., Desilets D., Karimian K., Tam T.F. (2003). 1,2,4-Thiadiazole: A novel cathepsin B inhibitor. Bioorg. Med. Chem..

[36] Vega-Teijido M.A., Maluf S.E.C., Bonturi C.R., Sambrano J.R., Ventura O.N. (2014). Theoretical insight into the mechanism for the inhibition of the cysteine protease cathepsin B by 1,2,4-thiadiazole derivatives. J. Mol. Model..

[37] Kumar R., Kumar V., Lee K.W. (2021). A computational drug repurposing approach in identifying the cephalosporin antibiotic and anti-hepatitis C drug derivatives for COVID-19 treatment. Comput. Biol. Med..

[38] Frampton J.E. (2013). Ceftaroline fosamil: A review of its use in the treatment of complicated skin and soft tissue infections and community-acquired pneumonia. Drugs.

[39] Laudano J.B. (2011). Ceftaroline fosamil: A new broad-spectrum cephalosporin. J. Antimicrob. Chemother..

[40] Zhanel G.G., Sniezek G., Schweizer F., Zelenitsky S., Lagacé-Wiens P.R.S., Rubinstein E., Gin A.S., Hoban D.J., Karlowsky J.A. (2009). Ceftaroline: A novel broad-spectrum cephalosporin with activity against meticillin-resistant staphylococcus aureus. Drugs.

[41] Morrissey I., Ge Y., Janes R. (2009). Activity of the new cephalosporin ceftaroline against bacteraemia isolates from patients with community-acquired pneumonia. Int. J. Antimicrob. Agents.

[42] Delgado C.P., Rocha J.B.T., Orian L., Bortoli M., Nogara P.A. (2022). In silico studies of Mpro and PLpro from SARS-CoV-2 and a new class of cephalosporin drugs containing 1,2,4-thiadiazole. Struct. Chem..

[43] Wishart D.S., Feunang Y.D., Guo A.C., Lo E.J., Marcu A., Grant J.R., Sajed T., Johnson D., Li C., Sayeeda (2018). DrugBank 5.0: A major update to the DrugBank database for 2018. Nucleic Acids Res..

[44] Riccobene T.A., Pushkin R., Jandourek A., Knebel W., Khariton T. (2016). Penetration of Ceftaroline into the Epithelial Lining Fluid of Healthy Adult Subjects. Antimicrob. Agents Chemother..

[45] Giacobbe D.R., Russo C., Martini V., Dettori S., Briano F., Mirabella M., Portuanato F., Dentone C., Giacomini M., Berruti M. (2021). Use of ceftaroline in hospitalized patients with and without COVID-19: A descriptive cross-sectional study. Antibiotics.

[46] Beigel J.H., Tomashek K.M., Dodd L.E., Mehta A.K., Zingman B.S., Kalil A.C., Hohmann E., Chu H.Y., Luetkemeyer M.D., Castilha D.L. (2020). Remdesivir for the Treatment of COVID-19—Final Report. N. Engl. J. Med..

[47] Furuta Y., Gowen B.B., Takahashi K., Smee D.F., Bernard D.L. (2013). Favipiravir (T-705), a novel viral RNA polymerase inhibitor. Antiviral Res..

[48] Hayden F.G., Lenk R.P., Epstein C., Kang L.L. (2024). Oral Favipiravir Exposure and Pharmacodynamic Effects in Adult Outpatients With Acute Influenza. J. Infect. Dis..

[49] Tekçe G., Arican M., Karaduman Z.O., Turhan Y., Sağlam S., Yücel M.O., Coşkun S.K., Tuncer C., Uludağ V. (2024). Radiologic and histopathologic effects of favipiravir and hydroxychloroquine on fracture healing in rats. Naunyn Schmiedebergs Arch. Pharmacol..

[50] Tam T., Leung-Toung R., Li W., Spino M., Karimian K. (2005). Medicinal Chemistry and Properties of 1,2,4-Thiadiazoles. Mini-Rev. Med. Chem..

[51] Trott O., Olson A.J. (2010). AutoDock Vina: Improving the speed and accuracy of docking with a new scoring function, efficient optimization and multithreading. J. Comput. Chem..

[52] Visualization—BIOVIA—Dassault Systèmes^®^. https://www.3ds.com/products-services/biovia/products/molecular-modeling-simulation/biovia-discovery-studio/visualization/.

[53] Fadlalla M., Ahmed M., Ali M., Elshiekh A.A., Yousef B.A. (2022). Molecular Docking as a Potential Approach in Repurposing Drugs Against COVID-19: A Systematic Review and Novel Pharmacophore Models. Curr. Pharmacol. Rep..

[54] Pettersen E.F., Goddar T.D., Huang C.C., Couch G.S., Greenblatt D.M., Meng E.C., Ferrin T.E. (2004). UCSF Chimera—A visualization system for exploratory research and analysis. J. Comput. Chem..

[55] How to Perform Flexible Docking Using Autodock Vina? —Bioinformatics Review. https://bioinformaticsreview.com/20201010/how-to-perform-flexible-docking-using-autodock-vina/.

[56] Case D.A., Aktulga H.M., Belfon K., Ben-Shalom I.Y., Berryman J.T., Brozell S.R., Cerutti D.S., Cheatham T.E., Cisneros G.A., Cruzeiro V.W.D. (2023). AmberTools. J. Chem. Inf. Model..

[57] Maier J.A., Martinez C., Kasavajhala K., Wickstrom L., Hauser K.E., Simmerling C. (2015). ff14SB: Improving the Accuracy of Protein Side Chain and Backbone Parameters from ff99SB. J. Chem. Theory Comput..

[58] Michaud-Agrawal N., Denning E.J., Woolf T.B., Beckstein O. (2011). MD Analysis: A toolkit for the analysis of molecular dynamics simulations. J. Comput. Chem..

[59] Andrade M.A., Mottin M., Sousa B.K.D.P., Barbosa J.A.R.G., dos Santos Azevedo C., Silva C.L., de Andrade M.G., Motta F.N., Maulay-Bailly C., Amand S. (2023). Identification of novel Zika virus NS3 protease inhibitors with different inhibition modes by integrative experimental and computational approaches. Biochimie..

[60] (2021). Laboratory Biosafety Guidance Related to Coronavirus Disease (COVID-19): Interim Guidance. https://www.who.int/publications/i/item/WHO-WPE-GIH-2021.1.

[61] Laboratory Biosafety Guidance Related to Coronavirus Disease (COVID-19). https://www.who.int/publications/i/item/laboratory-biosafety-guidance-related-to-coronavirus-disease-(covid-19).

[62] Dludla P.V., Jack B., Viragavan A., Pheiffer C., Johnson R., Louw J., Muller C.F. (2018). A dose-dependent effect of dimethyl sulfoxide on lipid content, cell viability and oxidative stress in 3T3-L1 adipocytes. Toxicol. Rep..

[63] Caleffi G.S., Rosa A.S., Souza L.G., Avelar J.L.S., Nascimento S.M.R., Almeida V.M., Tucci A.R., Ferreira V.N., Silva A.J.M., Santos-Filho O.A. (2023). Aurones: A Promising Scaffold to Inhibit SARS-CoV-2 Replication. J. Nat. Prod..

[64] Tucci A.R., Rosa R.M., Rosa A.S., Chaves O.A., Ferreira V.N.S., Oliveira T.K.F., Souza D.D.C., Borba N.R.R., Dornelles L., Rocha N.S. (2023). Antiviral Effect of 5′-Arylchalcogeno-3-aminothymidine Derivatives in SARS-CoV-2 Infection. Molecules.

[65] Zagórska A., Czopek A., Fryc M., Jończyk J. (2024). Inhibitors of SARS-CoV-2 Main Protease (Mpro) as Anti-Coronavirus Agents. Biomolecules.

[66] Alves M.H.M.E., Mahnke L.C., Macedo T.C., dos Santos Silva T.K., Carvalho Junior L.B. (2022). The enzymes in COVID-19: A review. Biochimie.

[67] Tan B., Zhang X., Ansari A., Jadhav P., Tan H., Li K., Chopra A., Ford A., Chi X., Ruiz F.X. (2024). Design of a SARS-CoV-2 papain-like protease inhibitor with antiviral efficacy in a mouse model. Science (1979).

[68] Antonopoulou I., Sapountzaki E., Rova U., Christakopoulos P. (2022). Inhibition of the main protease of SARS-CoV-2 (Mpro) by repurposing/designing drug-like substances and utilizing nature’s toolbox of bioactive compounds. Comput. Struct. Biotechnol. J..

[69] Yang K.S., Ma X.R., Alugubelli Y.R., Scott D., Vatanserver E.C., Drelich A.K., Sankaran B., Geng Z.Z., Blankenship L.R., Ward H.E. (2021). A Quick Route to Multiple Highly Potent SARS-CoV-2 Main Protease Inhibitors*. ChemMedChem.

[70] Shawky A.M., Almalki F.A., Alzahrani H.A., Abdalla A.N., Youssif B.G., Ibrahim N.A., Gamal M., El-Sherief H.A.M., Abdel-Fattah M.M., Hefny A.A. (2024). Covalent small-molecule inhibitors of SARS-CoV-2 Mpro: Insights into their design, classification, Biological Activity, and binding interactions. Eur. J. Med. Chem..

[71] Lockbaum G.J., Reyes A.C., Lee J.M., Tilvawala R., Nalivaika E.A., Ali A., Yilmaz N.K., Thompson P.R., Schiffer C.A. (2021). Crystal structure of sars-cov-2 main protease in complex with the non-covalent inhibitor ml188. Viruses.

[72] Han S.H., Goins C.M., Arya T., Shin W., Maw J., Hoopwe A., Sonawane D.P., Porter M.R., Bannister B.E., Crouch R.D. (2022). Structure-Based Optimization of ML300-Derived, Noncovalent Inhibitors Targeting the Severe Acute Respiratory Syndrome Coronavirus 3CL Protease (SARS-CoV-2 3CLpro). J. Med. Chem..

[73] Štekláč M., Zajaček D., Bučinský L. (2021). 3CLpro and PLpro affinity, a docking study to fight COVID19 based on 900 compounds from PubChem and literature. Are there new drugs to be found?. J. Mol. Struct..

[74] Koebel M.R., Cooper A., Schemadeke G., Jeon S., Narayan M., Sirimulla S. (2016). S···O and S···N Sulfur Bonding Interactions in Protein-Ligand Complexes: Empirical Considerations and Scoring Function. J. Chem. Inf. Model..

[75] Sanders B.C., Pokhrel S., Labbe A., Mathews I., Cooper I., Davidson R., Phillips G., Zhang Q., Neill H.O., Kaur M. (2023). Potent and selective covalent inhibition of the papain-like protease from SARS-CoV-2. Nat. Commun..

[76] Wang Q., Chen G., He J., Li J., Xiong M., Su H., Li M., Hu H., Xu Y. (2023). Structure-Based Design of Potent Peptidomimetic Inhibitors Covalently Targeting SARS-CoV-2 Papain-like Protease. Int. J. Mol. Sci..

[77] Jadhav P., Liang X., Ansari A., Tan B., Tan H., Li K., Chi X., Ford A., Ruiz F.X., Arnold E. (2025). Design of quinoline SARS-CoV-2 papain-like protease inhibitors as oral antiviral drug candidates. Nat. Commun..

[78] Huang S.Y. (2018). Comprehensive assessment of flexible-ligand docking algorithms: Current effectiveness and challenges. Brief. Bioinform..

[79] Stanzione F., Giangreco I., Cole J.C. (2021). Use of molecular docking computational tools in drug discovery. Prog. Med. Chem..

[80] Sargsyan K., Lin C., Chen T., Grauffel C., Chen Y., Yamg W., Yuan H.S., Lim C. (2020). Multi-targeting of functional cysteines in multiple conserved SARS-CoV-2 domains by clinically safe Zn-ejectors. Chem. Sci..

[81] Puhl A.C., Godoy A.S., Noske G.D., Nakamura A.M., Gawriljuk V.O., Fernandes R.S., Oliva G.O., Ekins S. (2023). Discovery of PLpro and Mpro Inhibitors for SARS-CoV-2. ACS Omega.

[82] Liu W., Wang J., Yue K., Hu Y., Liu X., Wang L., Wan S., Xu X. (2023). Discovery of new non-covalent and covalent inhibitors targeting SARS-CoV-2 papain-like protease and main protease. Bioorg. Chem..

[83] Narayanan A., Narwak M., Majowicz S.M., Varricchio C., Tones S.A., Ballatore C., Brancale A., Murakami K.S., Jose J. (2022). Identification of SARS-CoV-2 inhibitors targeting Mpro and PLpro using in-cell-protease assay. Commun. Biol..

[84] Chen H.-F., Hsueh P., Liu Y., Chen Y., Chang S., Wang W., Wu C., Tsai Y., Liu Y., Su W. (2022). Disulfiram blocked cell entry of SARS-CoV-2 via inhibiting the interaction of spike protein and ACE2. Am. J. Cancer Res..

[85] Qiao Z., Wei N., Jin L., Zhang H., Luo J., Zhang Y., Wang K. (2021). The Mpro structure-based modifications of ebselen derivatives for improved antiviral activity against SARS-CoV-2 virus. Bioorg. Chem..

[86] Paxlovid|Therapeutic Goods Administration (TGA). https://www.tga.gov.au/resources/auspmd/paxlovid.

[87] Atmar R.L., Finch N. (2022). New Perspectives on Antimicrobial Agents: Molnupiravir and Nirmatrelvir/Ritonavir for Treatment of COVID-19. Antimicrob. Agents Chemother..

[88] Ringer A.L., Senenko A., Sherrill C.D. (2007). Models of S/π interactions in protein structures: Comparison of the H2S–benzene complex with PDB data. Protein Sci..

[89] Reid K.S.C., Lindley P.F., Thornton J.M. (1985). Sulphur-aromatic interactions in proteins. FEBS Lett..

[90] Justo Arevalo S., Castillo-Chavez A., Calampa C.S.U., Sifuentes S.Z., Huallpa C., Bianchi G.L., Casas R.G. (2023). What do we know about the function of SARS-CoV-2 proteins?. Front. Immunol..

[91] Kakavandi S., Zare I., VaezJalali M., Azarian M., Akbari A., Farani M.R., Zalpoor H., Hajikhani B. (2023). Structural and non-structural proteins in SARS-CoV-2: Potential aspects to COVID-19 treatment or prevention of progression of related diseases. Cell Commun. Signal..

[92] Jackson C.B., Farzan M., Chen B., Choe H. (2022). Mechanisms of SARS-CoV-2 entry into cells. Nat. Rev. Mol. Cell Biol..

[93] Ho H.P.T., Vo D.N.K., Lin T., Hung J., Chiu Y., Tsai M. (2022). Ganoderma microsporum immunomodulatory protein acts as a multifunctional broad-spectrum antiviral against SARS-CoV-2 by interfering virus binding to the host cells and spike-mediated cell fusion. Biomed. Pharmacother..

[94] Zhang Q.Y., Li J., Zhang Y., Zhang Z., Li X., Zhang H., Deng C., Yang F., Xu Y., Zhang B. (2024). Identification of fangchinoline as a broad-spectrum enterovirus inhibitor through reporter virus based high-content screening. Virol. Sin..

[95] Roche K.L., Remiszewski S., Todd M.J., Kulp J.L., Tang L., Welsh A.V., Barry A.P., De C., Reiley W.W., Whal A. (2023). An allosteric inhibitor of sirtuin 2 deacetylase activity exhibits broad-spectrum antiviral activity. J. Clin. Investig..

[96] Wang Y., Zhang D., Du P.G., Zhao P.J., Jin Y., Fu S., Gao L., Cheng Z., Lu Q., Hu Y. (2020). Remdesivir in adults with severe COVID-19: A randomised, double-blind, placebo-controlled, multicentre trial. Lancet.

[97] Chen P., Nirula A., Heller B., Robert M.D., Gottieb R.L., Boscia J., Morris J., Huhn G., Cardona J., Mochela B. (2021). SARS-CoV-2 Neutralizing Antibody LY-CoV555 in Outpatients with COVID-19. N. Engl. J. Med..

[98] Nhean S., Varela M.E., Nguyen Y., Juarez A., Huynh T., Udeh D., Tseng A.L. (2021). COVID-19: A Review of Potential Treatments (Corticosteroids, Remdesivir, Tocilizumab, Bamlanivimab/Etesevimab, and Casirivimab/Imdevimab) and Pharmacological Considerations. J. Pharm. Pract..

[99] Mahase E. (2021). COVID-19: Pfizer’s paxlovid is 89% effective in patients at risk of serious illness, company reports. BMJ.

[100] Buchynskyi M., Oksenych V., Kamyshna I., Kamyshnyi O. (2024). Exploring Paxlovid Efficacy in COVID-19 Patients with MAFLD: Insights from a Single-Center Prospective Cohort Study. Viruses.

[101] Bartha F.A., Juhász N., Marzban S., Han R., Röst G. (2022). In Silico Evaluation of Paxlovid’s Pharmacometrics for SARS-CoV-2: A Multiscale Approach. Viruses.

[102] Angus D.C., Berry S., Lewis R.J., Al-Beidh F., Arabi Y., Bentum-Puijk W., Bhimani Z., Bonten M., Broglio K., Brunkhorst F. (2020). The remap-cap (Randomized embedded multifactorial adaptive platform for community-acquired pneumonia) Study rationale and design. Ann. Am. Thorac. Soc..

[103] Study Details | Randomized, Embedded, Multifactorial Adaptive Platform Trial for Community- Acquired Pneumonia|ClinicalTrials.gov. https://clinicaltrials.gov/study/NCT02735707.

[104] Soriano A., Bassetti M., Gogos C., Ferry T., Pablo R., Ansari W., Kantecki M., Schweikert B., Luna G., Blasi F. (2024). Ceftaroline fosamil treatment patterns and outcomes in adults with community-acquired pneumonia: A real-world multinational, retrospective study. JAC Antimicrob. Resist..

