# Supplementary material for: In Silico and In Vitro Studies of the Approved Antibiotic Ceftaroline Fosamil and Its Metabolites as Inhibitors of SARS-CoV-2 Replication
Source: Viruses. 2025 Mar 28;17(4):491. doi: 10.3390/v17040491 (PMC12031345; doi:10.3390/v17040491)
Supplement: Supplementary file 1 [file viruses-17-00491-s001.zip › viruses-3480002-supplementary.pdf]

In Silico and *In Vitro* Studies of the Approved Antibiotic Ceftaroline Fosamil and its Metabolites as Inhibitors of SARS-CoV-2 Replication

Cássia Delgado[a], Pablo Nogara[a,b*], Milene D. Miranda[c,d*], Alice S. Rosa[c,d], Vivian Neuza S. Ferreira[c], Luisa Tozatto Batista[c], Thamara Kelcya F.[c,d], Folorunsho Bright Omage[e], Flávia Motta[f], Izabela Marques Bastos[f], Laura Orian[g], João Batista Teixeira Rocha[a].

[a] Departamento de Bioquímica e Biologia Molecular, Centro de Ciências Naturais e Exatas, Universidade Federal de Santa Maria, Santa Maria, RS, Brazil. *Corresponding author: pbnogara@gmail.com

[b] Instituto Federal de Educação, Ciência e Tecnologia Sul-rio-grandense (IFSul), Bagé, RS, Brazil.

[c] Laboratório de Morfologia e Morfogênese Viral, Instituto Oswaldo Cruz, Fundação Oswaldo Cruz, Rio de Janeiro 21041-250, RJ, Brazil. *Corresponding author: mmiranda@ioc.fiocruz.br

[d] Programa de Pós-Graduação em Biologia Celular e Molecular, Instituto Oswaldo Cruz, Fundação Oswaldo Cruz, Rio de Janeiro 21041-250, RJ, Brazil.

[f] Laboratório de interface patógeno-hospedeiro, Departamento de Biologia Celular, Universidade de Brasília (UnB), Brasilia 70910-900, DF, Brazil.

[e] Biological Chemistry Laboratory, Department of Organic Chemistry, Institute of Chemistry, University of Campinas (UNICAMP), Campinas, São Paulo, Brazil

[g] Dipartimento di Scuenze Chimiche, Università degli Studi di Padova, Via Marzolo 1, 35129 Padova, Italy.

Summary

Figure S1 3
Figure S2 4
Figure S3 5
Figure S4 6
Figure S5 7
Figure S6 8
Figure S7 9
Figure S8 10
Figure S9 11
Figure S10 12
Figure S11 13

Figure 12 14
Figure 13 15
Table 14 15
Table 15 16

1. Docking analysis results

1.1. M^{pro} docking simulations

When analyzed the distances between Cys145 S···S 1,2,4-thiadiazole in the conformer characterized by the largest negative ΔG binding energy, the distance analysis in the semi-flexible simulation, demonstrated more favorable S···S distances for CF (4.3 Å) and M2-metabolite (5.6 Å), the binding free energies in these conformers analyses of M^{pro} presented values from -8.6 to -6.7 (ΔG, kcal·mol^{-1}) (**Figure S4**) (**Table S14-$^{c}M^{pro}$**).

Regarding the analyses of the conformer with the most favorable S···S interaction distances between Cys145 and 1,2,4-thiadiazole, in the semi-flexible simulation, the M1-metabolite and the open-M1H-metabolite presented the most favorable distances, 3.6 Å and 3.4 Å, and the binding free energies in these conformers analyses of M^{pro} presented values from -7.9 and -7.4 (ΔG, kcal·mol^{-1}), respectively (**Figure S3**) (**Table S15-$^{c}M^{pro}$**).

1.2. PL^{pro} docking simulations

The analysis in the semi-flexible simulation for the conformer characterized by the largest negative ΔG binding energy, demonstrated less favorable S···S distances for CF and their derivatives metabolites, compared to M^{pro} analysis in the same molecular docking methodology, with values varying between 6.7 Å to 12.5 Å and binding free energies values between -7.3 to -5.7(ΔG, kcal·mol^{-1}) (**Table S14. $^{d}PL^{pro}$**) (**Figure S8**).

The semi-flexible simulation in the conformer with the most favorable S···S interaction distances between Cys111 S···S 1,2,4-thiadiazole moiety, the M1-metabolite and the M1H-metabolite presented the most favorable distance, 3.6 Å and 3.7 Å and binding free energies values between -5.7 to -6.0 kcal·mol^{-1}, respectively (**Table S15. $^{d}PL^{pro}$**) (**Figure S7**).

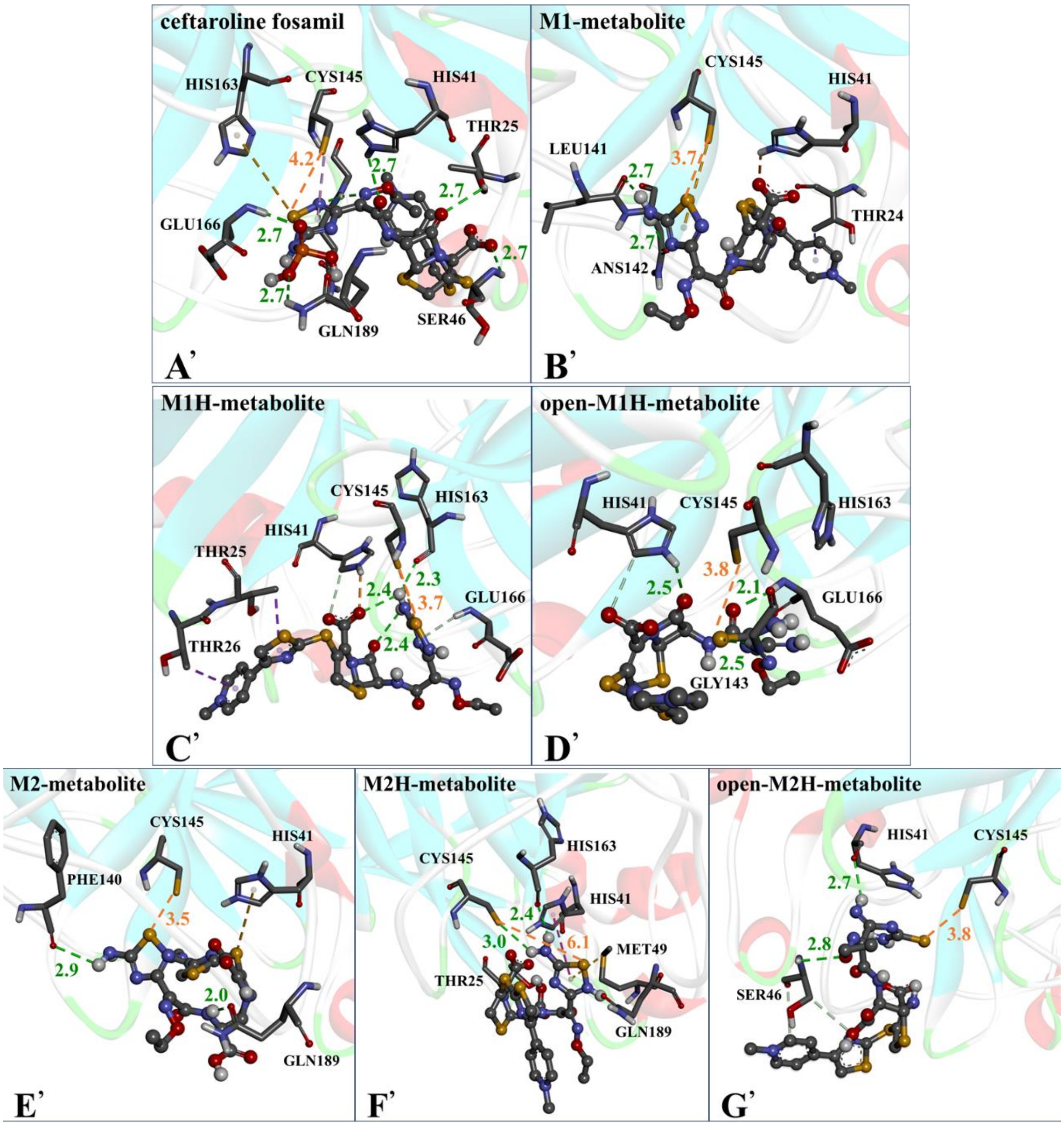

Figure S1. Rigid docking focusing in the active site with Cys and His charged- M^{pro} with ceftaroline fosamil and its metabolites in the favorable S···S interaction distances. **A**) ceftaroline fosamil. **B**) M1-metabolite. **C**) M1H-metabolite. **D**) open-M1H-metabolite. **E)** M2-metabolite. **F)** M2H-metabolite. **G)** open-M2H-metabolite. Distances are shown in Å.

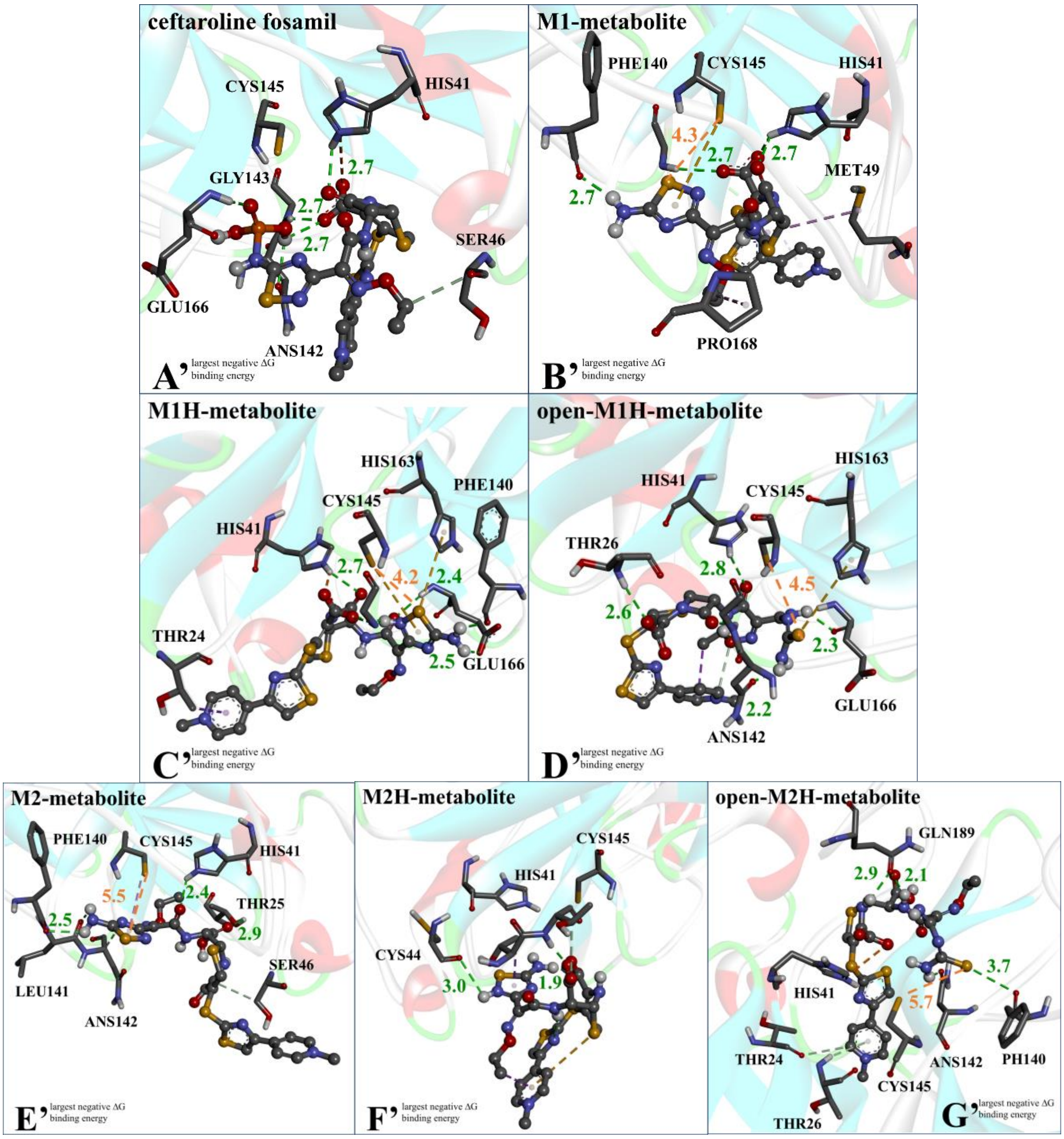

Figure S2. Rigid docking focusing in the active site with Cys and His charged- M^{pro} with ceftaroline fosamil and its metabolites in the largest negative ΔG binding energy. **A**) ceftaroline fosamil. **B**) M1-metabolite. **C**) M1H-metabolite. **D**) open-M1H-metabolite. **E)** M2-metabolite. **F)** M2H-metabolite. **G)** open-M2H-metabolite. Distances are shown in Å.

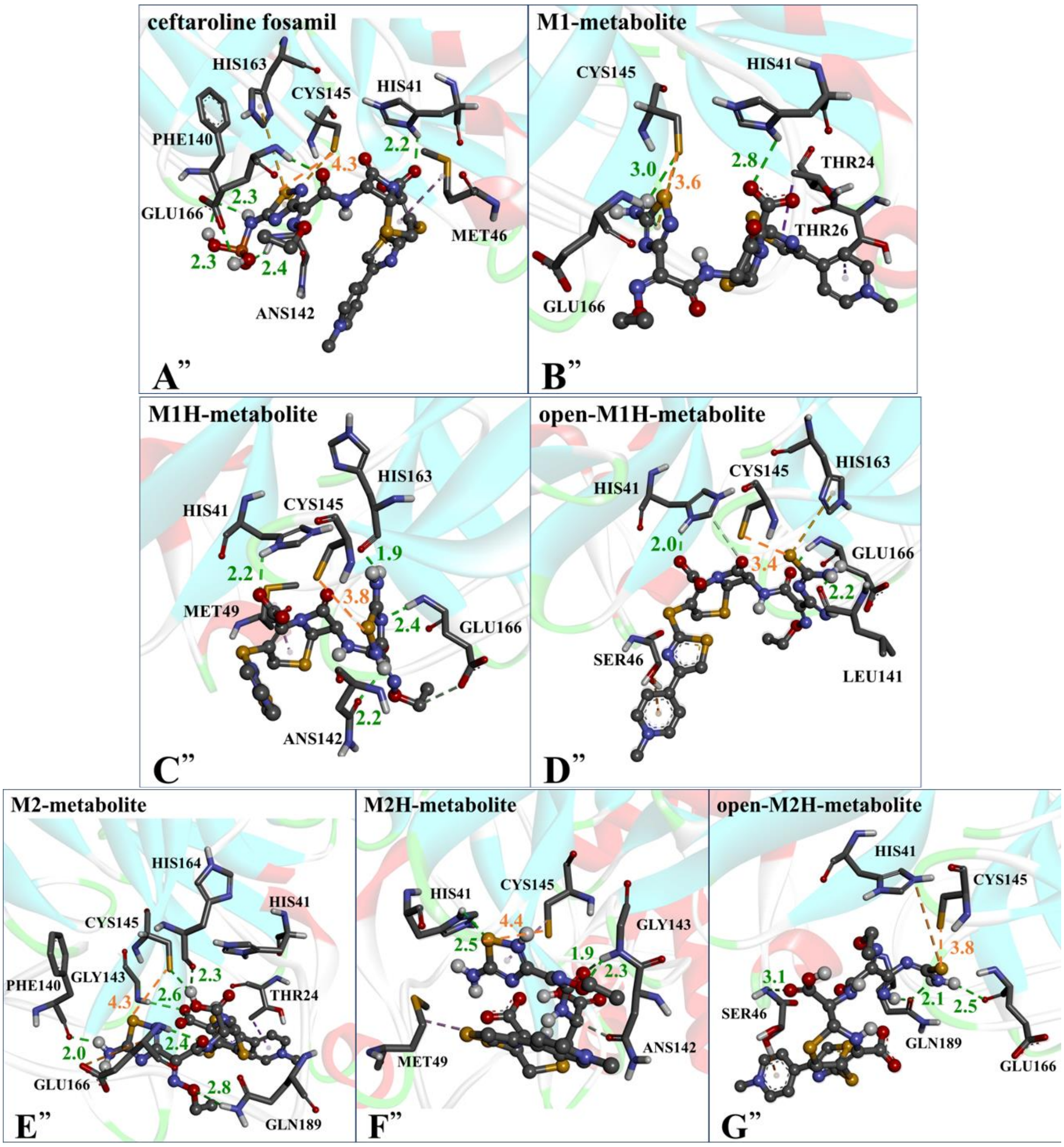

Figure S3. Semi-flexible docking- M^{pro} with ceftaroline fosamil and its metabolites in the favorable S⋯S interaction distances. **A**) ceftaroline fosamil. **B**) M1-metabolite. **C**) M1H-metabolite. **D**) open-M1H-metabolite. **E)** M2-metabolite. **F)** M2H-metabolite. **G)** open-M2H-metabolite. Distances are shown in Å.

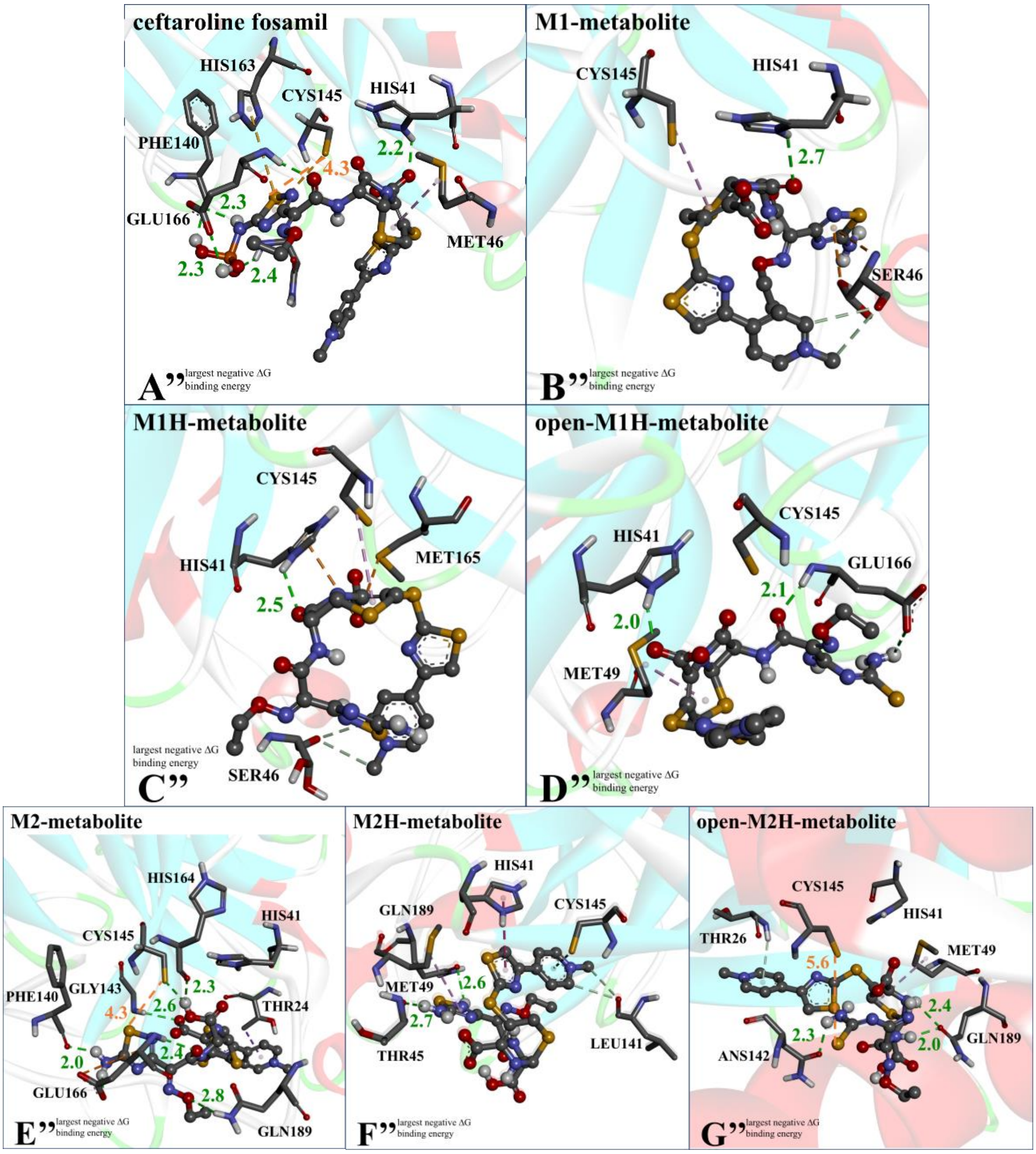

Figure S4. Semi-flexible docking- M^{pro} with ceftaroline fosamil and its metabolites in the largest negative ΔG binding energy. **A**) ceftaroline fosamil. **B**) M1-metabolite. **C**) M1H-metabolite. **D**) open-M1H-metabolite. **E)** M2-metabolite. **F)** M2H-metabolite. **G)** open-M2H-metabolite. Distances are shown in Å.

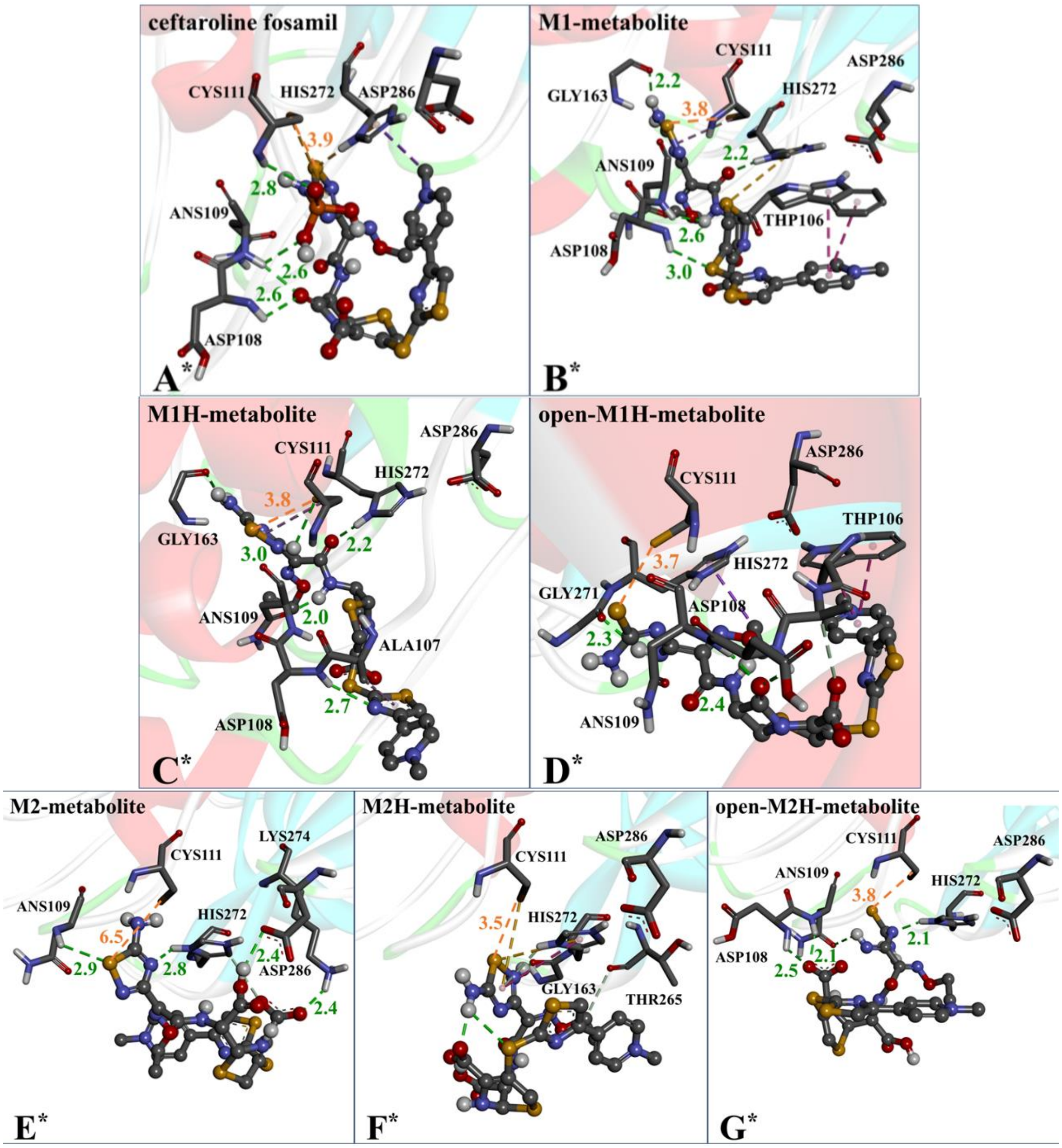

Figure S5. Rigid docking focusing in the active site with Cys and His charged- PL^{pro} with ceftaroline fosamil and its metabolites in the favorable S⋯S interaction distances. **A**) ceftaroline fosamil. **B**) M1-metabolite. **C**) M1H-metabolite. **D**) open-M1H-metabolite. **E)** M2-metabolite. **F)** M2H-metabolite. **G)** open-M2H-metabolite. Distances are shown in Å.

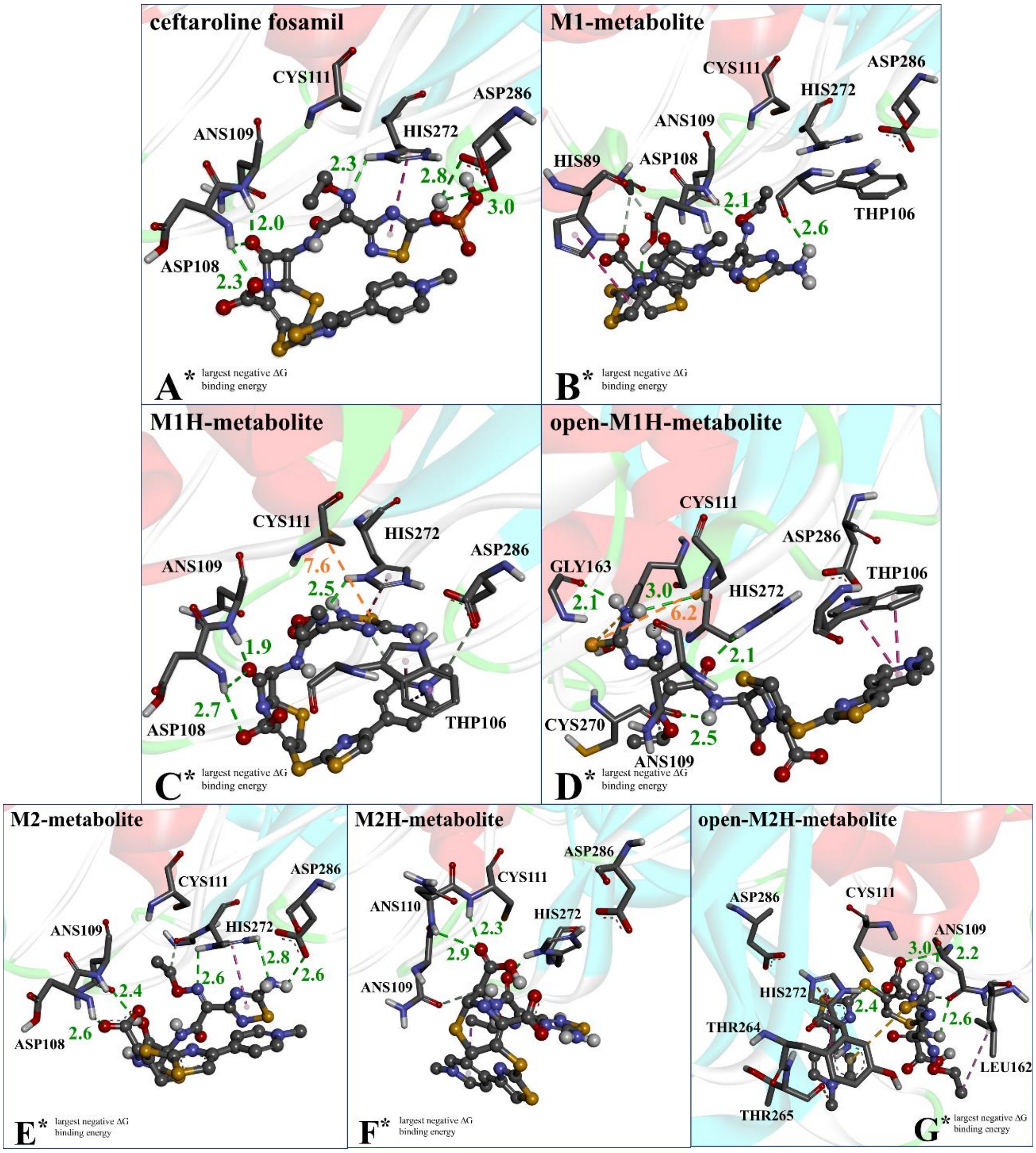

Figure S6. Rigid docking focusing in the active site with Cys and His charged- PL^{pro} with ceftaroline fosamil and its metabolites in the largest negative ΔG binding energy. **A**) ceftaroline fosamil. **B**) M1-metabolite. **C**) M1H-metabolite. **D**) open-M1H-metabolite. **E)** M2-metabolite. **F)** M2H-metabolite. **G)** open-M2H-metabolite. Distances are shown in Å.

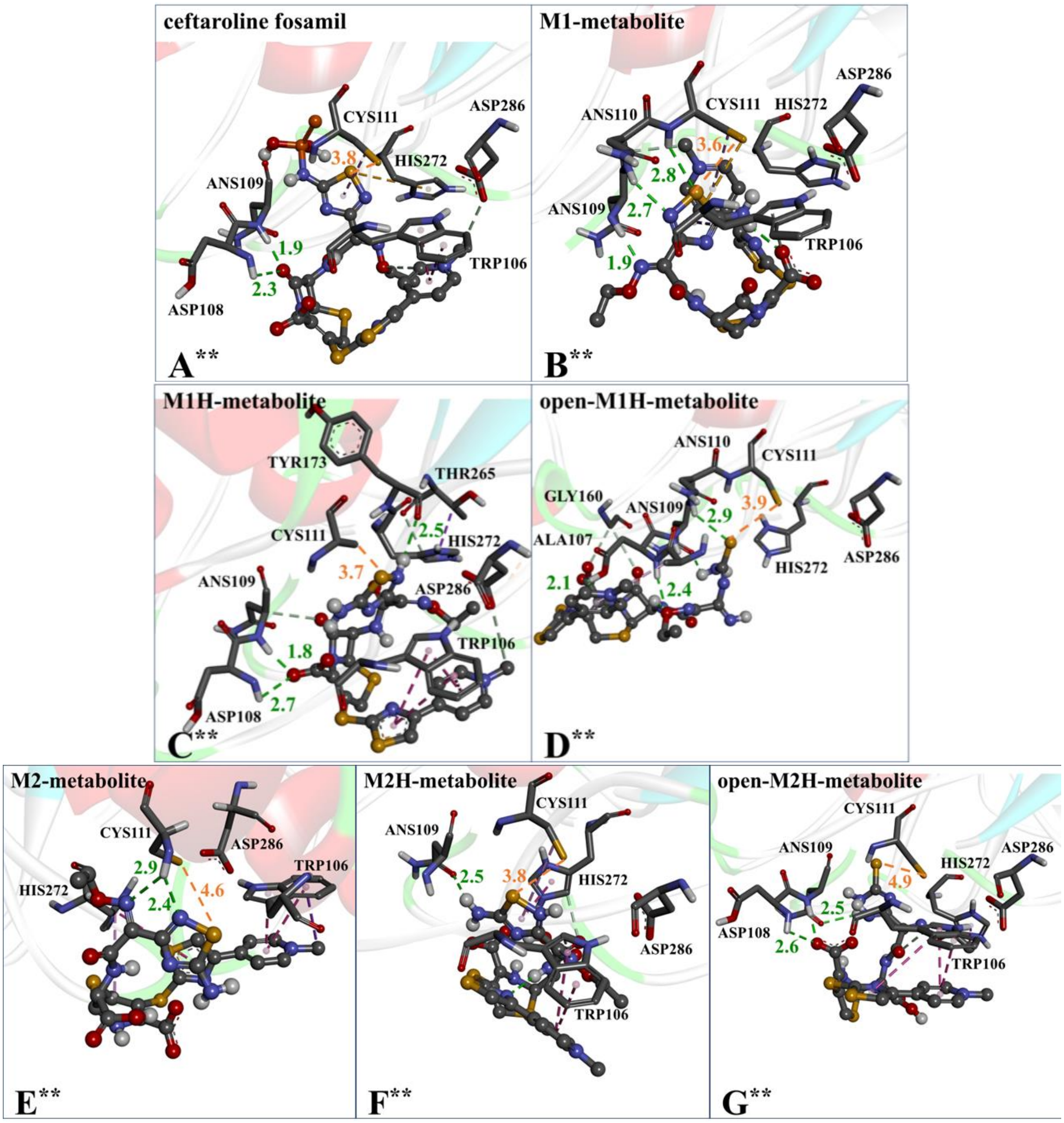

Figure S7. Semi-flexible docking- PL^{pro} with ceftaroline fosamil and its metabolites in the favorable S⋯S interaction distances. **A**) ceftaroline fosamil. **B**) M1-metabolite. **C**) M1H-metabolite. **D**) open-M1H-metabolite. **E)** M2-metabolite. **F)** M2H-metabolite. **G)** open-M2H-metabolite. Distances are shown in Å.

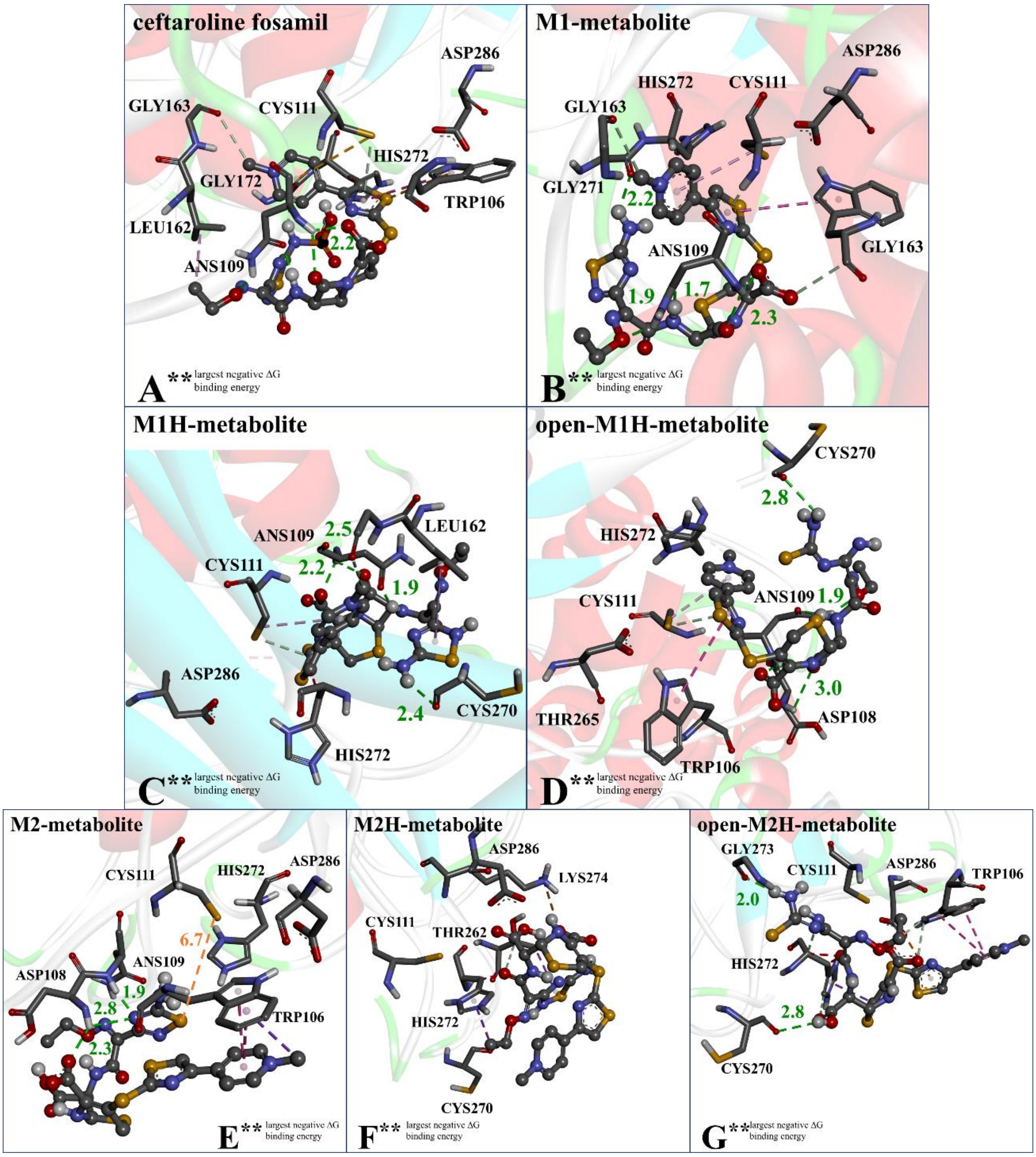

Figure S8. Semi-flexible docking- PL^{pro} with ceftaroline fosamil and its metabolites in the largest negative ΔG binding energy. **A**) ceftaroline fosamil. **B**) M1-metabolite. **C**) M1H-metabolite. **D**) open-M1H-metabolite. **E)** M2-metabolite. **F)** M2H-metabolite. **G)** open-M2H-metabolite. Distances are shown in Å.

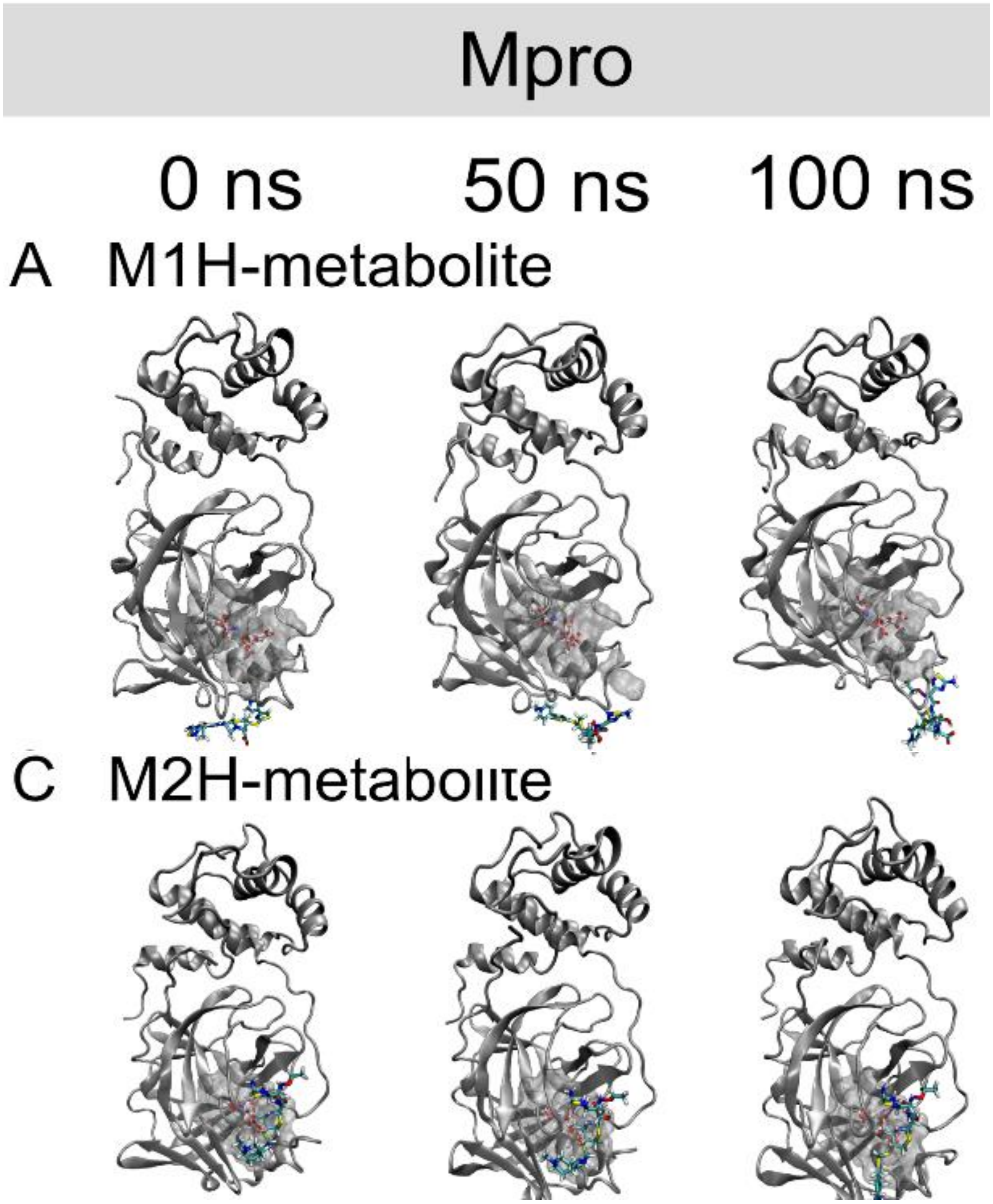

Figure S9. Comparative timeframe evolution of the trajectories for each of the four metabolites analyzed in the conformational dynamics within the protein's for M^{pro} binding pocket, over 0 ns to 100 ns production time. The simulation area for each protein is represented by a gray cloud, with only the catalytic dyad and triad in each enzyme's binding pocket highlighted (M^{pro} Cys145, His41) using a ball-and-stick representation. The metabolites M1H, M2H, open-M1H and open-M2H depicted as colorful sticks.

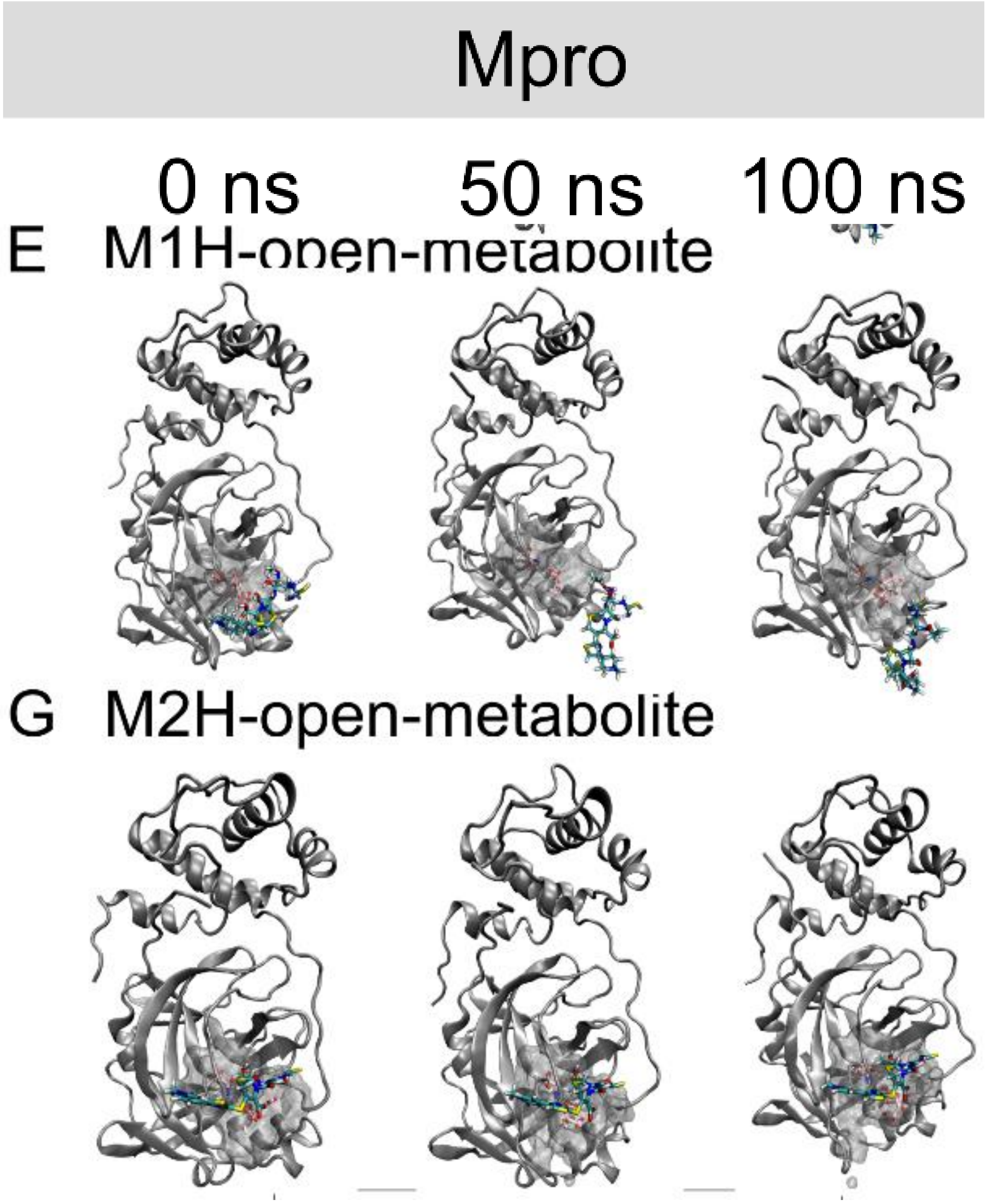

Figure S10. Comparative timeframe evolution of the trajectories for each of the four metabolites analyzed in the conformational dynamics within the protein's for M^{pro} binding pocket, over 0 ns to 100 ns production time. The simulation area for each protein is represented by a gray cloud, with only the catalytic dyad and triad in each enzyme's binding pocket highlighted (M^{pro} Cys145, His41) using a ball-and-stick representation. The metabolites M1H, M2H, open-M1H and open-M2H depicted as colorful sticks**.**

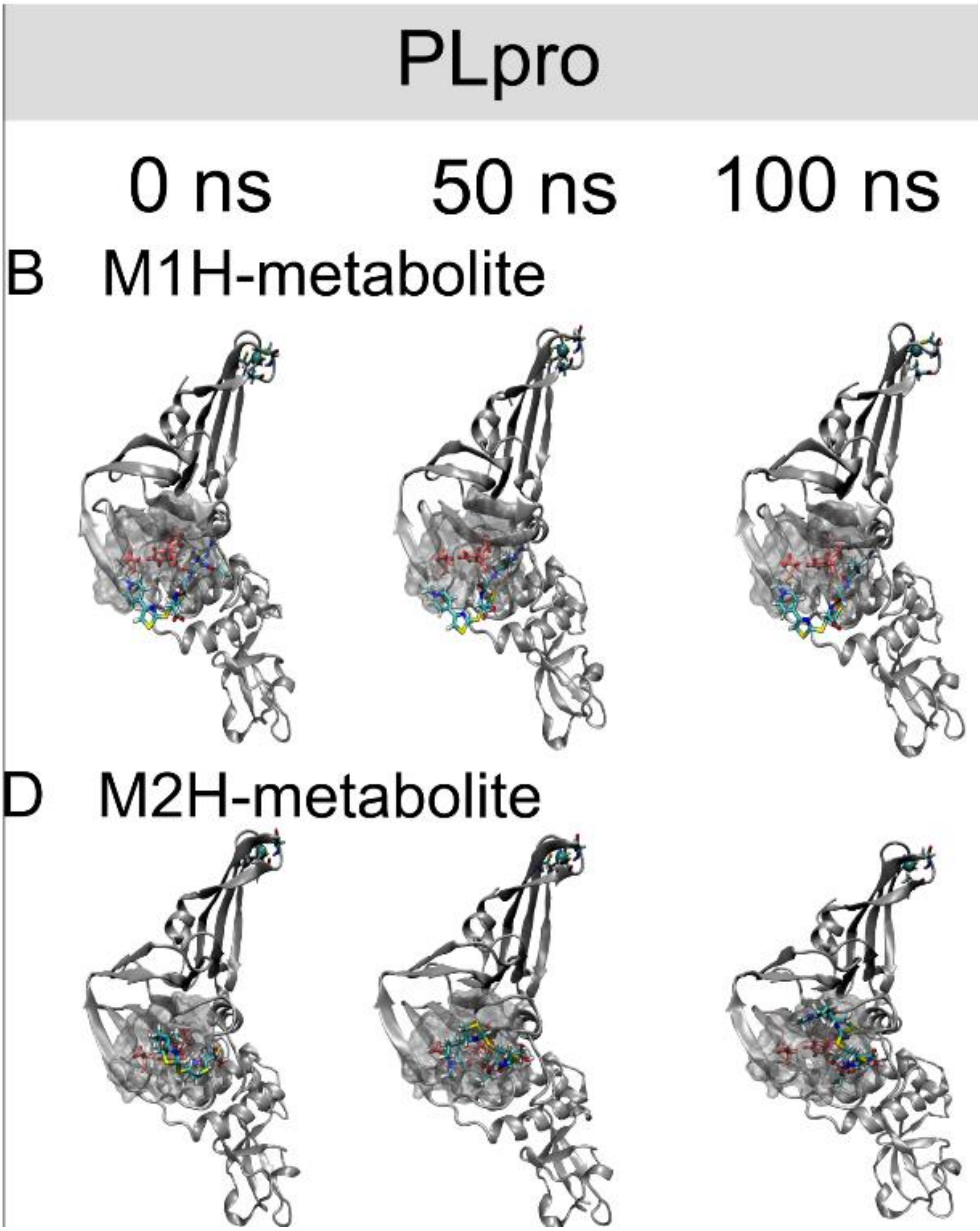

Figure S11. Comparative timeframe evolution of the trajectories for each of the four metabolites analyzed in the conformational dynamics within the protein's for PL^{pro} binding pocket, over 0 ns to 100 ns production time. The simulation area for each protein is represented by a gray cloud, with only the catalytic dyad and triad in each enzyme's binding pocket highlighted (Cys111, His272, Asp286) using a ball-and-stick representation. The metabolites M1H, M2H, open-M1H and open-M2H depicted as colorful sticks**.**

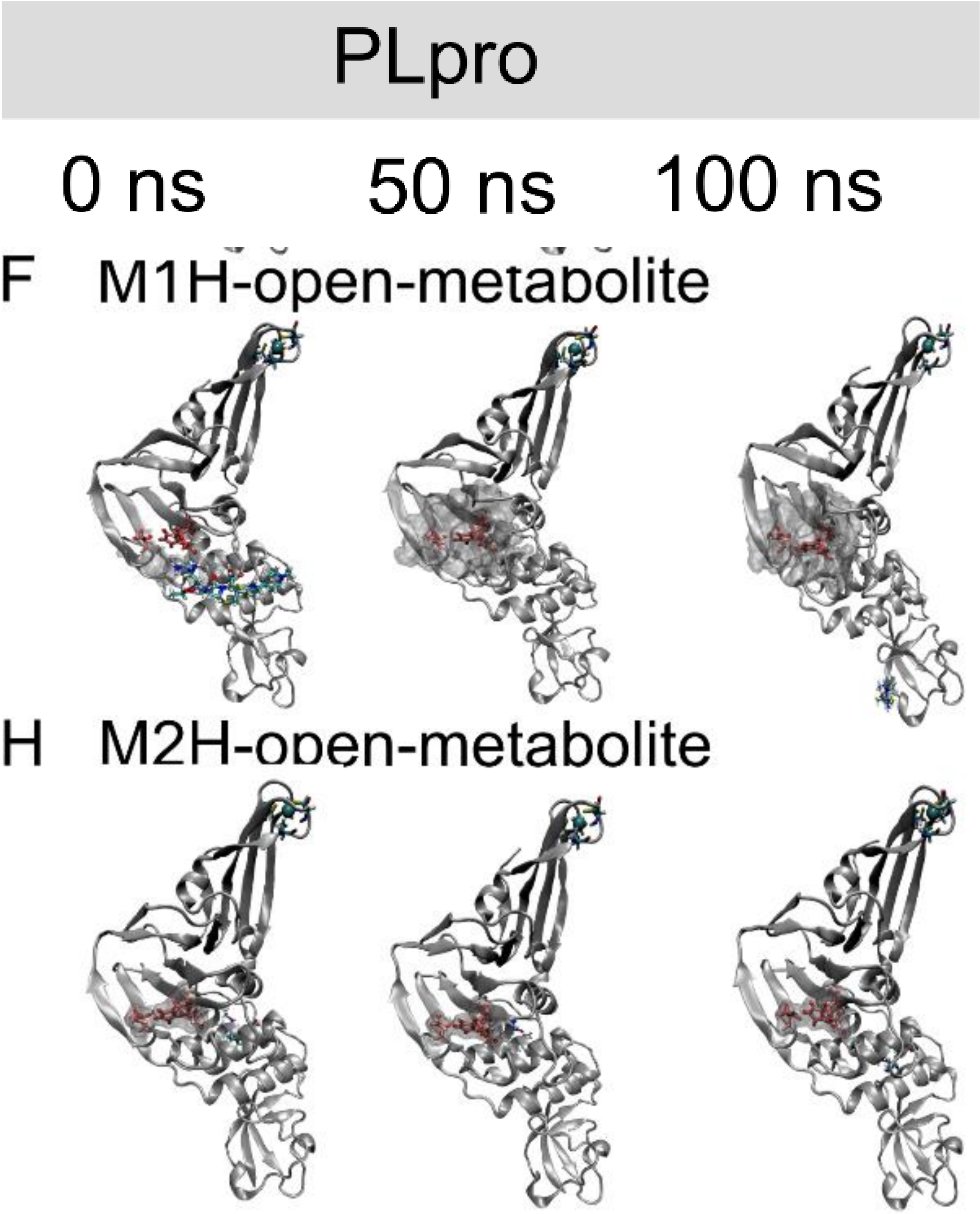

Figure S12. Comparative timeframe evolution of the trajectories for each of the four metabolites analyzed in the conformational dynamics within the protein's for PL^{pro} binding pocket, over 0 ns to 100 ns production time. The simulation area for each protein is represented by a gray cloud, with only the catalytic dyad and triad in each enzyme's binding pocket highlighted (Cys111, His272, Asp286) using a ball-and-stick representation. The metabolites M1H, M2H, open-M1H and open-M2H depicted as colorful sticks**.**

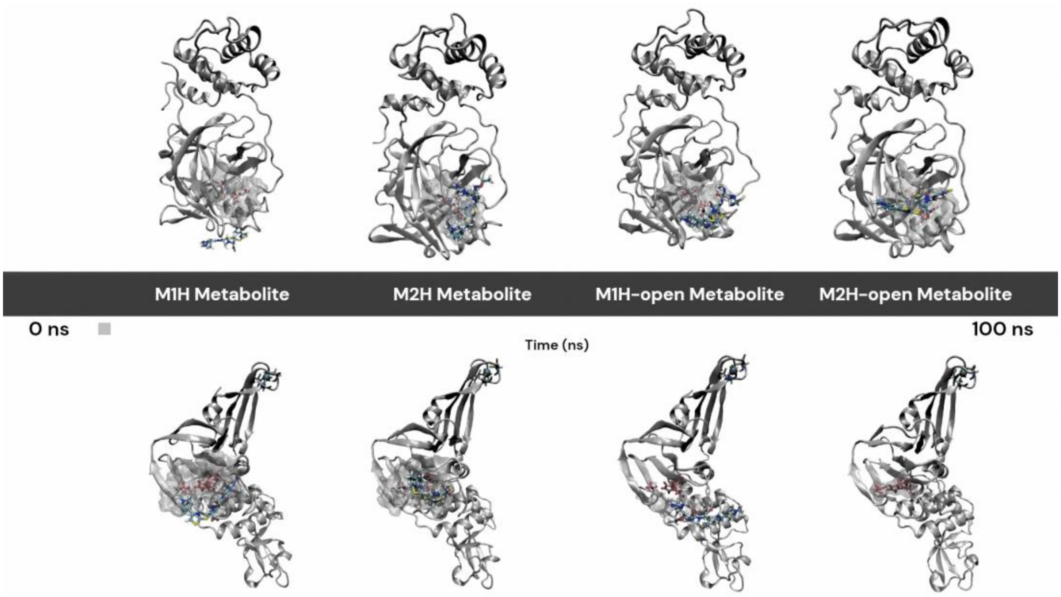

Figure S13. Timeframe evolution of the trajectories for each of the four metabolites analyzed in the conformational dynamics within the protein's for M^{pro} (top) and PL^{pro} (below) binding pocket, over 0 ns to 100 ns production time. The simulation area for each protein is represented by a gray cloud, with only the catalytic dyad and triad in each enzyme's binding pocket highlighted (M^{pro} Cys145, His41/ PL^{pro} Cys111, His272, Asp286) using a ball-and-stick representation. The metabolites M1H, M2H, open-M1H and open-M2H depicted as colorful sticks. Better resolution and details in the link.

https://drive.google.com/file/d/1xN-MKvk-ypipSUg10MGKSQYO3A1eO8ul/view?usp=sharing

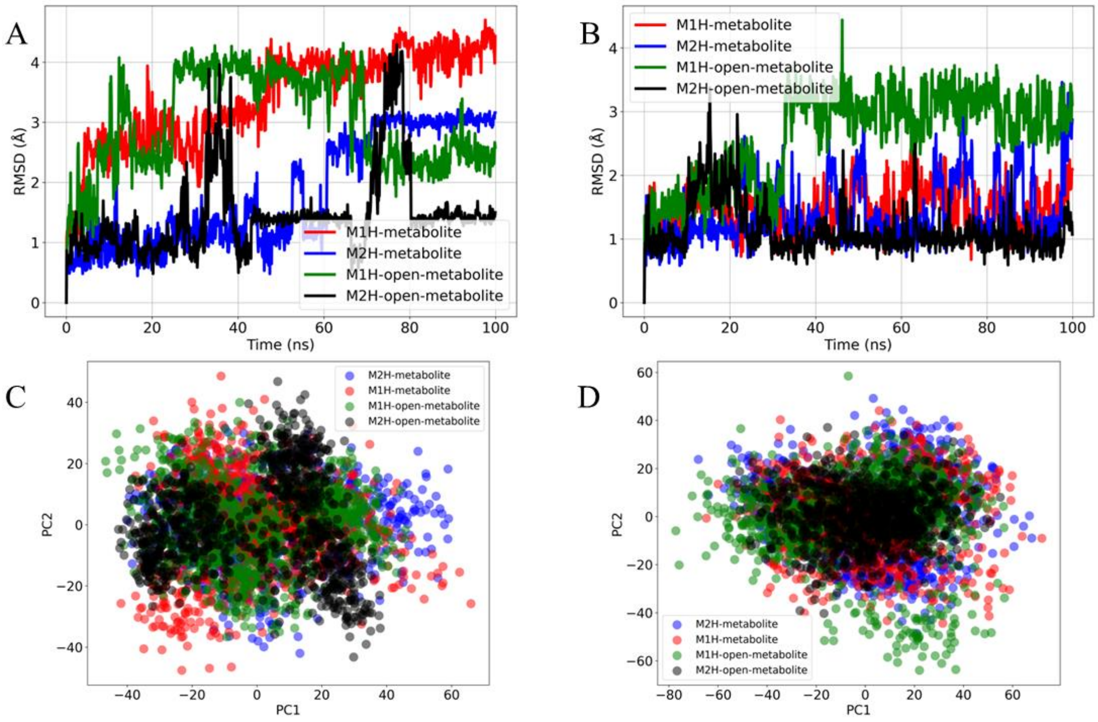

Figure S14. RMSD values are calculated with respect to each metabolite's conformation at 0ns production time (A) and (B) are the RMSD of the M^{pro} and PL^{pro} metabolites alone in the complexes. (C) M^{pro} and (D) PL^{pro} Principal component analysis (PCA) plot showing the metabolite complex conformations. Color-coded

by metabolic state: M2H (blue), M1H (red), open-M1H (green), and open-M2H (black). The spread along the PC1 and PC2 axes indicates variance in conformation.

Table S15. Predicted binding free energies (ΔG, kcal·mol^{-1}) between M^{pro} andPL^{pro}, flexible binding site, with CF and metabolites for the conformer presenting the largest negative ΔG.

	$^{c}M^{pro}$		$^{d}PL^{pro}$	
Molecule	**ΔG**	dist. (Å) S^{-}*···S (Cys 145)	**ΔG**	dist. (Å) S^{-}*···S (Cys 111)
ceftaroline fosamil	-8.6	4.3	-7.3	11.2
M1- metabolite	-8.4	11.9	-6.7	10.8
M1H- metabolite	-8.6	10.0	-6.4	11.0
open- M1H- metabolite	-8.2	10.8	-6.8	7.8
M2- metabolite	-7.3	4.3	-6.0	6.7
M2H- metabolite	-7.8	9.7	-6.3	12.5
open-M2H- metabolite	-7.9	5.6	-5.7	8.2

c- M^{pro} Semi-flexible docking with Cys and His charged, d- M^{pro} Semi-flexible docking with Cys and His charged. The S^{-}*···S indicates the distance (in Å) of the sulfur atom of thiolate of Cys145 and Cys111, to the sulfur atom from 1,2,4-thiadiazole heterocycle. The green, yellow, and red colors indicate a favorable, intermediate, and less favorable interaction, respectively.

Table S16. Predicted binding free energies (ΔG, kcal·mol^{-1}) between M^{pro} and PL^{pro}, flexible binding site, with CF and metabolites with the conformer presenting the most favorable S···S interaction distances.

	$^{c}M^{pro}$		$^{d}PL^{pro}$	
Molecule	**ΔG**	dist. (Å) S^{-}*···S (Cys 145)	**ΔG**	dist. (Å) S^{-}*···S (Cys 111)
ceftaroline fosamil	-8.3	4.3	-6.2	3.8
M1- metabolite	-7.9	3.6	-5.7	3.6
M1H- metabolite	-8.4	3.8	-6.0	3.7
open- M1H- metabolite	-7.4	3.4	-6.3	3.9
M2- metabolite	-7.3	4.3	5.7	4.6
M2H- metabolite	-7.4	4.4	-5.4	3.8
open-M2H- metabolite	-7.4	3.8	-5.5	4.9

c- M^{pro} Semi-flexible docking with Cys and His charged, d- M^{pro} Semi-flexible docking with Cys and His charged. The S^{-}*···S indicates the distance (in Å) of the sulfur atom of thiolate of Cys145 and Cys111, to the sulfur atom from 1,2,4-thiadiazole heterocycle. The green, yellow, and red colors indicate a favorable, intermediate, and less favorable interaction, respectively.
